# Glucocorticoid-Responsive Transcription Factor Krüppel-Like Factor 9 Regulates *fkbp5* and Metabolism

**DOI:** 10.3389/fcell.2021.727037

**Published:** 2021-10-06

**Authors:** Ian M. Gans, Janelle Grendler, Remy Babich, Nishad Jayasundara, James A. Coffman

**Affiliations:** ^1^MDI Biological Laboratory, Bar Harbor, ME, United States; ^2^Graduate School of Biomedical Science and Engineering, University of Maine, Orono, ME, United States; ^3^The School of Marine Sciences, University of Maine, Orono, ME, United States; ^4^Nicholas School of the Environment, Duke University, Durham, NC, United States

**Keywords:** glucocorticoid receptor, Krüppel-like factor 9, *fkbp5*, cortisol, metabolism, zebrafish, RNA-seq, gene expression

## Abstract

Krüppel-like factor 9 (Klf9) is a feedforward regulator of glucocorticoid receptor (GR) signaling. Here we show that in zebrafish *klf9* is expressed with GR-dependent oscillatory dynamics in synchrony with *fkbp5*, a GR target that encodes a negative feedback regulator of GR signaling. We found that *fkbp5* transcript levels are elevated in *klf9*^–/–^ mutants and that Klf9 associates with chromatin at the *fkbp5* promoter, which becomes hyperacetylated in *klf9*^–/^*^–^* mutants, suggesting that the GR regulates *fkbp5* via an incoherent feedforward loop with *klf9*. As both the GR and Fkbp5 are known to regulate metabolism, we asked how loss of Klf9 affects metabolic rate and gene expression. We found that *klf9*^–/–^ mutants have a decreased oxygen consumption rate (OCR) and upregulate glycolytic genes, the promoter regions of which are enriched for potential Klf9 binding motifs. Our results suggest that Klf9 functions downstream of the GR to regulate cellular glucocorticoid responsivity and metabolic homeostasis.

## Introduction

Glucocorticoids, the terminal hormonal output of the vertebrate hypothalamus-pituitary-adrenal (HPA) axis, function to maintain homeostasis by regulating diverse aspects of physiology, including metabolism and the immune system. Endogenous glucocorticoids (cortisol in humans and fish, corticosterone in rodents) are crucial for mounting physiological responses to stimuli that are either predictable (e.g., day/night cycles, feeding schedule) or unexpected (e.g., during the fight/flight response to acute threat). Basal circulating glucocorticoid (GC) levels oscillate with a circadian rhythm, peaking in the hours just before an animal’s active phase (early morning in diurnal species, evening in nocturnal species). This circadian fluctuation is produced by amplitude modulation of shorter ultradian (i.e., ∼hourly) pulses of hormone secretion (Veldhuis et al., 1989; Jasper and Engeland, 1991; Stavreva et al., 2009). Ultradian GC pulses cause cycles of glucocorticoid receptor (GR) activation and association with chromatin that produce ultradian pulses of transcription (Stavreva et al., 2009). Acute stressors also activate the HPA axis and pulsed GC release, and the magnitude of the physiological response depends on whether the stressor occurs during the rising or falling phase of the ultradian rhythm (Windle et al., 1998).

Because the GR is ubiquitously expressed and binds thousands of genomic sites in a given tissue (Sacta et al., 2016), changes in circulating GC levels produce broad changes in gene expression, and hence in physiology and behavior. Temporal gene regulation is one way in which organisms optimize usage of resources (Liu et al., 1995; Klevecz et al., 2004; Lloyd and Murray, 2005; Tu et al., 2005), and conditions that disrupt GC dynamics—e.g., chronic/repeated stress, interference with circadian cues, or Cushing’s Disease (hypercortisolemia)—are associated with multi-systemic disorders, including immune, psychological and metabolic syndromes (Silverman and Sternberg, 2012). We have previously reported that treatment of zebrafish larvae with chronic 1 μM cortisol leads to aberrant immune gene expression as well as long-term effects on the dynamics of the hypothalamus-pituitary-interrenal (HPI, equivalent to the mammalian HPA) axis and immune gene expression (Hartig et al., 2016, 2020).

GC signaling dynamics are controlled by negative feedback at both systemic and intracellular levels. Systemically, negative feedback of circulating GC on the pituitary is key to the generation of ultradian GC pulses (Walker et al., 2010, 2012; Spiga et al., 2011). Within cells, a negative feedback loop exists between the GR and its co-chaperone Fkbp5: transcription of *fkbp5* is activated by the GR and translated Fkbp5 inactivates the GR by sequestering it in the cytoplasm, increasing resistance to further activation by GC (Denny et al., 2000; Scammell et al., 2001). Elevated *FKBP5* expression contributes to HPA dysfunction and associated diseases (Binder et al., 2004; Binder, 2009; Klengel et al., 2012; Li et al., 2020), including diabetes and obesity (Pereira et al., 2014; Pena et al., 2020). However, our knowledge of *fkbp5* transcriptional regulation is incomplete.

Krüppel-like Factors (KLFs) are zinc-finger transcription factors that co-regulate nuclear receptor signaling (Knoedler and Denver, 2014) and fine-tune transcription through competitive binding to regulatory DNA (Ilsley et al., 2017). *Klf9* is a GR target that is ubiquitously expressed, including in the brain (Bonett et al., 2009; Bagamasbad et al., 2012; Juszczak and Stankiewicz, 2018) wherein it directs a maladaptive response to chronic stress (Besnard et al., 2018), and in the liver where its overexpression contributes to hyperglycemia (Cui et al., 2019). GR*-klf9* feedforward regulation was documented in human skin and murine macrophages (Chinenov et al., 2014; Lili et al., 2019), and by our lab in zebrafish (Gans et al., 2020), a model organism well-suited for studies of GC signaling (Dickmeis et al., 2007; Griffiths et al., 2012; Nesan and Vijayan, 2013). Here we extend our previous report that Klf9 mediates transcriptomic effects of chronic GC exposure by asking if Klf9 contributes to the regulation of *fkbp5* and metabolism, key targets of GC signaling. We found that *klf9* and *fkbp5* are synchronously expressed with GR-dependent dynamics that differ from those of other GC-responsive genes; that *fkbp5* activity is elevated in *klf9*^–/–^ mutants; and that Klf9 protein interacts with chromatin at the *fkbp5* promoter. We also show that metabolism is altered in *klf9*^–/–^ mutants, as indicated by decreased oxygen consumption rate (OCR) and upregulation of glycolytic genes.

## Materials and Methods

### Zebrafish Strains, Husbandry, and Embryo Treatments

The AB strain was used for all genetic modifications. Husbandry and procedures were carried out as described previously (Hartig et al., 2016) and in the Supplementary Methods. All animal procedures were approved by the Institutional Animal Care and Use Committee (IACUC) of the MDI Biological Laboratory, and all methods were performed in accordance with the relevant guidelines and regulations.

The Klf9-AMtag line was created using CRISPR-Cas9 and homology-directed repair as described in the Supplementary Methods, using a donor sequence encoding the AM epitope tag (Supplementary Figure 10) recognized by the Active Motif anti-AM antibody (Active Motif, Carlsbad, CA).

### Analysis of Gene Expression

Quantitative reverse transcription and polymerase chain reaction (qRT-PCR) was carried out as described previously (Hartig et al., 2016) and in the Supplementary Methods. For time-course experiments, each timepoint sample consisted of 3–6 larvae cultured in an individual Petri dish, and the dishes were arranged in the incubator so as to minimize disturbance during sampling. Sinusoid models were fit to time-course expression data using the equation y = m + a^∗^exp(-b^∗^x)^∗^sin(c^∗^(x-d)), where m = MESOR, a = amplitude, b = decay rate, c = 2^∗^pi^∗^frequency, and d = phase shift. Non-linear least squares regression was performed using either the *nls* function in the R Stats package or in Microsoft Excel using the Solver function (Kemmer and Keller, 2010); both methods generated the same solutions. Initial values for constants were chosen to generate a reasonable qualitative fit before iterative fitting programs were run. When generating random data sets and model fits (see section “Results”), initial constants were set to the best fit of data combined from both conditions (either VEH- and CORT-treated, or wildtype and *klf9*^–/–^, depending on the experiment).

RNA-seq data were obtained from our previous study (Gans et al., 2020). The generation and analysis of the data are described in the Supplementary Methods.

The NanoString PlexSet assay for 24 probe sets (21 genes of interest plus 3 reference genes) was performed according to the manufacturer’s instructions (NanoString Technologies, Seattle, WA) and as described in the Supplementary Methods. Probe sets were designed by NanoString with direction and approval of the authors (see Supplementary Table 1). Normalized counts were exported from the NanoString nSolver program (Geiss et al., 2008), and heat maps were generated from Z-transformed log2 counts data using the *heatmap.2* function in gplots 3.1.1 package (Warnes et al., 2020) in R. Hierarchical clustering was determined using the *hclust* function in the R stats package and the “complete” method with distance between rows calculated by the *dist* function and Euclidean method.

### Cortisol Measurement

Cortisol was assayed using the Neogen Cortisol ELISA kit, as described previously (Hartig et al., 2016) and in the Supplementary Methods.

### Chromatin Immunoprecipitation

Chromatin Immunoprecipitation (ChIP) was performed as described by Lindeman et al. (2009) and in the Supplementary Methods.

### Oxygen Consumption Rate

Oxygen consumption rate was analyzed using an XF96^e^ Extracellular Flux Analyzer (Agilent Technologies) as described in the Supplementary Methods. The first data point is typically inconsistent in these runs and was therefore excluded from the analysis. The remaining 11 data points were imported to GraphPad Prism 8.0 (GraphPad Software, San Diego, CA, United States) and plotted as a function of time (Supplementary Figure 12A). Linear regression of time-series data was used to determine the slope (rate of change in OCR over time) and Y-intercept, which is indicative of basal metabolic rate. Statistical significance between treatment groups for the slopes and Y-intercepts were calculated by two-factor ANOVA (treatment and genetic background as the two variable, Supplementary Figure 12B) followed by Šídák’s multiple comparisons test (GraphPad Prism 8.0). A possible confound that we cannot exclude is that CORT and VEH treatment solutions could have differences in oxygen solubility.

## Results

### *Klf9* and *fkbp5* Share Synchronous and Glucocorticoid Receptor-Dependent Temporal Expression Dynamics

**FIGURE 1:**
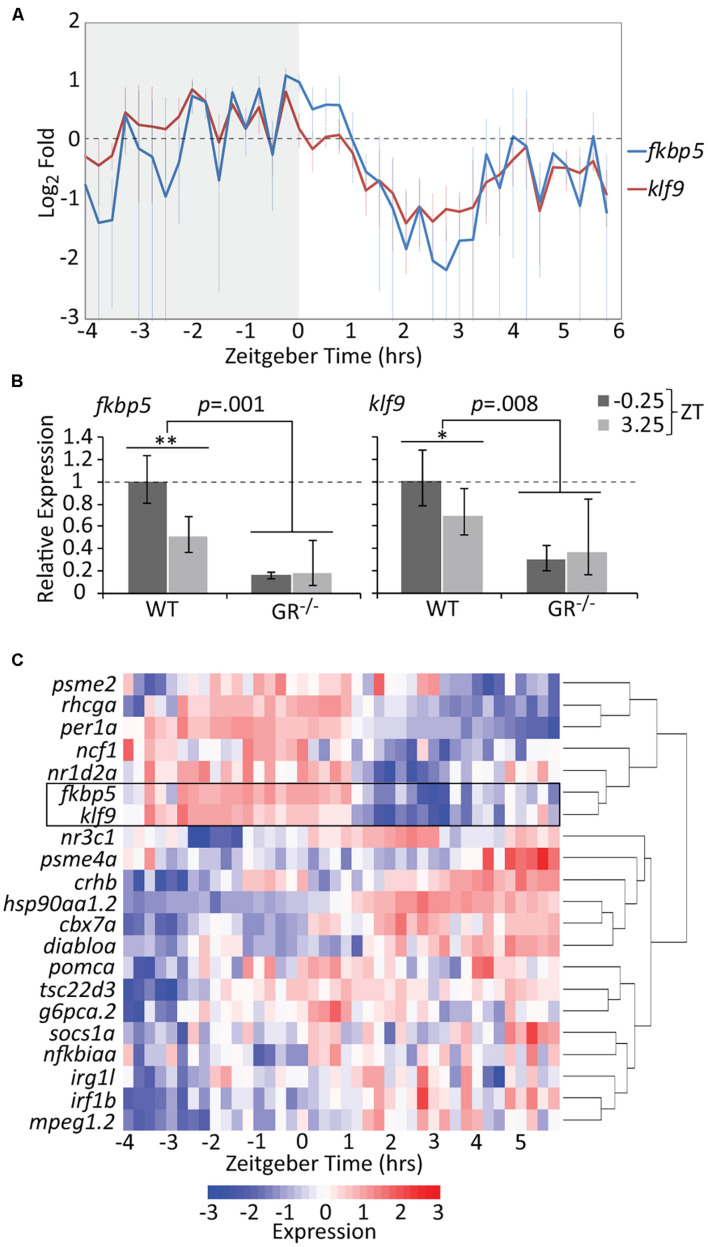
The temporal activities of the GR targets *klf9* and *fkbp5* are dynamic and synchronous. **(A)** Average relative levels of *klf9* and *fkbp5* transcripts in three biological replicates of pooled (*n* = 3–6) 5 dpf larvae snap-frozen every 15 min from −4 to 6 h zeitgeber time (ZT). Error bars are standard error of the mean of three biological replicates. **(B)** Relative expression of *klf9* and *fkbp5* in WT and *nr3c1*^–/–^ (GR^369–^, Gans et al., 2020) mutant larvae at −0.25 and +3.25 ZT, time points that, respectively, correspond to the activity peak and nadir of both genes in WT larvae. Error bars represent 95% confidence intervals of three biological replicates of pooled larvae (*n* = 9 per sample). Significance calculated by two-factor (time and genotype) ANOVA, and one-tailed *t*-tests to assess the effect of time within each genotype; ^∗∗^*p* = 0.01; ^∗^*p* = 0.05. **(C)** Heat map of expression of *klf9*, *fkbp5*, and additional targets/regulators of glucocorticoid (GC) signaling as measured on the NanoString platform. Gene panel includes well-known glucocorticoid receptor (GR) targets as well as targets identified by our lab as consistently over-expressed in chronic CORT treated (Gans et al., 2020). Counts data are normalized to reference genes (*actb2*, *rpl13a*, and *eif5a*) and scaled within each gene to normalize for different absolute levels of expression.

Treatment of zebrafish embryos with chronic 1 μM cortisol (CORT) produces a modest but significant elevation in whole-body cortisol and GR activity at 5 days post-fertilization (dpf) (Hartig et al., 2016). The CORT-treated larvae upregulate immune-related genes, an effect dependent on both the GR and Klf9 (Gans et al., 2020), and give rise to adults in which *fkbp5* and *klf9* transcripts are persistently elevated in blood cells on average, although not in every instance (Hartig et al., 2020). We hypothesized that such inconsistency could be an artifact of measuring dynamic gene expression with insufficient temporal resolution, and therefore undertook high-density time-course sampling to measure *fkbp5* and *klf9* expression in 5 dpf larvae, by which stage the HPI axis is fully developed (Alsop and Vijayan, 2008) and both genes are actively transcribed (Supplementary Figure 1). Although bulk analysis of larval gene expression lacks spatial resolution, we reasoned that such an analysis would be informative since both *fkbp5* and *klf9* are ubiquitously expressed (Thisse and Thisse, 2004; Zhang et al., 2017), and transcription in peripheral zebrafish tissues is highly entrained to circadian rhythms via GC signaling (Frøland Steindal and Whitmore, 2019; Morbiato et al., 2019). For the time-courses, care was taken not to disturb larvae during sampling (see section “Materials and Methods”) to minimize stress-responsive expression. The results revealed that *klf9* and *fkbp5* are expressed with synchronous oscillations on both the circadian and ultradian time scales (Figure 1A and Supplementary Figure 1C). Transcript levels of both genes peaked just prior to zeitgeber time 0 (ZT 0, corresponding to lights-on in the incubator), falling sharply thereafter. The data for both genes could be fit by non-linear regression to sinusoidal models, with wavelengths of 8.2 and 9.5 h for *fkbp5* and *klf9* respectively (ANOVA *p* < 0.0001, Supplementary Figure 2).

Since the 10-h sampling window was too narrow to draw conclusions about circadian periodicity, we measured expression of *klf9*, *fkbp5* and the circadian gene *per1a* over a 24-h period. While *per1a* had a prototypical 24-h oscillation, *klf9* and *fkbp5* had a shorter periodicity and lower amplitude oscillation (Supplementary Figure 3). Nevertheless, transcript levels of all three genes peaked synchronously just before ZT 0 and dropped precipitously thereafter (Supplementary Figure 3). We interpret this as coordination of GR activity with the circadian clock, as whole-larva cortisol levels show a similar diurnal drop (Supplementary Figure 4) and GCs drive circadian cell cycle and metabolic rhythms in zebrafish larvae (Dickmeis et al., 2007; Weger et al., 2016). We verified the GR-dependence and diurnal dynamics of *klf9* and *fkbp5* activity by comparing transcript levels in wild-type larvae and GR^–/–^ mutants at ZT −0.25 and ZT 3. In the GR^–/–^ mutants, both genes were lowly and flatly expressed, being significantly under-expressed at the wildtype (WT) expression peak of ZT −0.25 and remaining below WT at the 3.25 ZT expression nadir (Figure 1B).

To determine if the temporal dynamics shared by *klf9* and *fkbp5* are common to GC-responsive genes in general we re-measured one replicate (40 samples of pooled larvae) of our time-course using the NanoString platform and a probe set (Supplementary Table 1) representing 21 genes known to be direct GR targets and/or that we have experimentally identified as consistently responding to CORT treatment, including *klf9* and *fkbp5* (Gans et al., 2020). Hierarchical clustering of these NanoString data distinguished two main time-dependent clusters of genes (Figure 1C). The tight correlation between *klf9* and *fkbp5* was underscored by their clustering together on their own branch, which in turn clustered with other genes whose expression peaked in early morning hours, including circadian regulators *per1a* and *nr1d2a*, as well as *rhcga* and *ncf1*. Conversely, for the second main cluster, which included the GR target *tsc22d3* as well as several immune genes, expression was low in the early morning when endogenous CORT is high, then rose after ZT 0.

**FIGURE 2:**
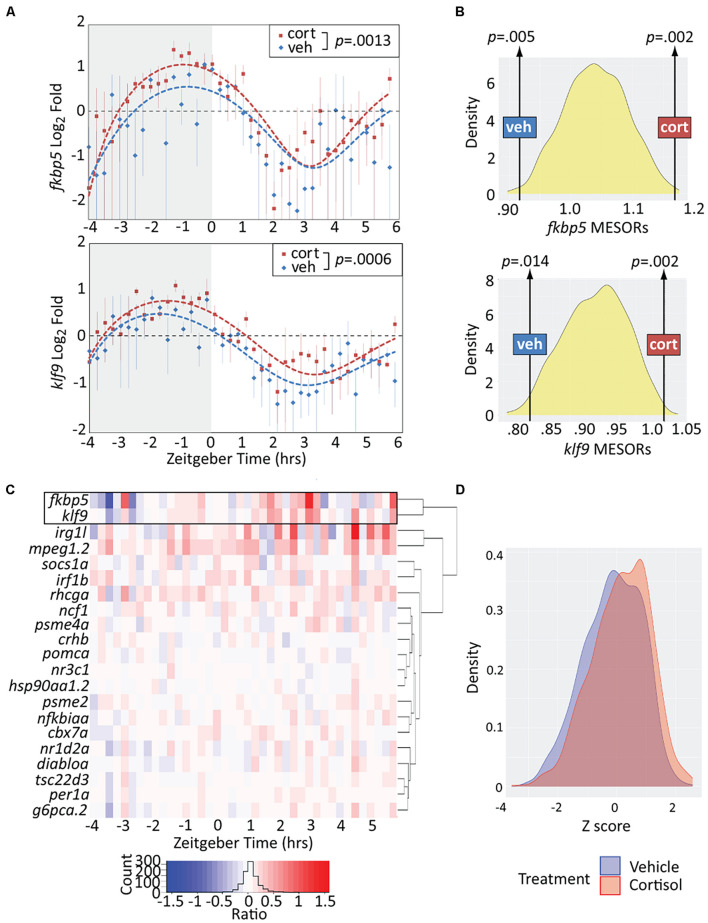
Chronic cortisol treatment similarly upregulates *klf9* and *fkbp5*. **(A)** Sinusoid models fit to *fkbp5* (top) and *klf9* (bottom) qPCR relative expression data. Each data point represents the average of three biological replicates of pooled larvae (3–6 larvae per pooled sample; VEH-treated datapoints are the same as in Figure 1). *P*-values calculated using paired *t*-test (data paired at each timepoint). Fit of the model to the data was tested by ANOVA (*P* < 0.0001 in each case, see Supplementary Figure 2). **(B)** Distributions of Midline Estimated Statistics Of Rhythmicity (MESORs, a rhythm-adjusted mean) of datasets generated by randomly selecting expression data from VEH or CORT samples at each timepoint in experiments shown in panel **(A)**. MESORs of VEH and CORT datasets sit at either extreme. **(C)** Heat map of the expression ratio in CORT/VEH samples (log2 transformed) of *klf9*, *fkbp5*, and other targets of glucocorticoid (GC) signaling in our Nanostring data set. **(D)** Density plot of Z-scored expression of all genes measured with Nanostring indicates an overall increase in expression due to chronic CORT treatment.

We next assessed the effect of chronic CORT exposure, shown by our previous studies to elevate *klf9* transcript levels at the 5 dpf mid-morning (∼ZT 3) timepoint when samples were collected (Hartig et al., 2016; Gans et al., 2020). Overall, the treatment resulted in a significant elevation of *klf9* and *fkbp5* transcripts across all timepoints (*p* < 0.005, paired *t*-tests), albeit with instances of lower levels at some timepoints (Figure 2A). To rigorously test whether the exposure significantly increased the average expression of these genes we also computationally generated 1,000 time series in which either VEH or CORT measurements were randomly selected at each timepoint in the experimental data. We then fit sinusoid models to each randomized time series. Each sinusoid equation includes five constants (see section “Materials and Methods”), including the Midline Estimating Statistic Of Rhythm (MESOR, a rhythm-adjusted mean value). For both *fkbp5* and *klf9*, the MESORs of models fit to either VEH or CORT experimental data were located at opposite extremes of approximately normal distributions of MESORs from all randomized models (*p* < 0.02, Figure 2B), providing confirmation that chronic CORT exposure produced a statistically significant increase in *klf9* and *fkbp5* transcript levels.

Finally, to determine how the response of *klf9* and *fkbp5* to chronic CORT compares to that of the other GR targets in our NanoString panel we calculated the expression ratio of CORT- to VEH-treated samples at each timepoint for each gene. The CORT response of *klf9* and *fkbp5* was correlated, clustering separately from that of all other genes and manifesting a stronger effect than most (Figure 2C). The immune genes *mpeg1.2* and *irg1l* responded similarly in magnitude, but with different timing (rising rather than falling after ZT 0). The overall effect of the treatment across all genes was a subtle increase in transcript levels (Figure 2D).

### Krüppel-Like Factor 9 Negatively Regulates *fkbp5* and Binds *fkbp5* Promoter-Proximal Chromatin

**FIGURE 3:**
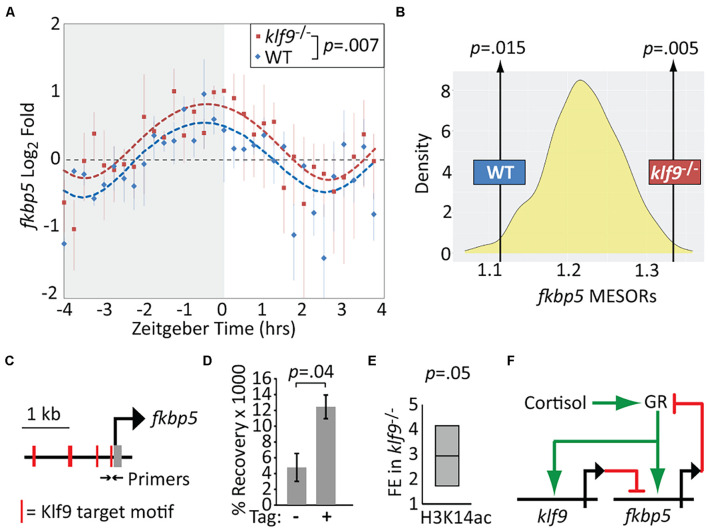
Krüppel-like factor 9 (Klf9) regulates *fkbp5*. **(A)** Sinusoid modeling of *fkbp5* time-course data obtained by quantitative reverse transcription and polymerase chain reaction (qRT-PCR) of RNA extracted from wildtype (WT) and *klf9*^–/–^ larvae. Each data point represents the average of three biological replicates of pooled larvae. *P*-value calculated by paired *t*-test (data paired at each timepoint). Error bars represent standard error of the mean. Model fits tested by ANOVA (*P* < 0.0001, see Supplementary Figure 4). **(B)** Distribution of Midline Estimated Statistics Of Rhythmicity (MESORs) of datasets generated by random sampling as described in Figure 2 and Methods. MESORs of measured WT and *klf9*^–/–^ data sit at either extreme. **(C)** Schematic of putative Klf9 target motifs in the *fkbp5* promoter region. Black arrows indicate sites of primers used in panels **(D,E)**. **(D)** Recovery of *fkbp5* promoter DNA from WT (-) and Klf9-AM tagged (+) larvae by ChIP with anti-AM tag antibody. **(E)** Fold enrichment (FE) of *fkbp5* promoter DNA recovered in *klf9*^–/–^ mutants with ChIP using antibody to acetyled-H3K14. **(F)** Proposed gene regulatory circuit containing the glucocorticoid receptor (GR), *fkbp5*, and *klf9.* Activation is denoted by green arrows, repression by red lines.

We performed additional time-courses to compare *fkbp5* mRNA levels in WT and *klf9*^–/–^ larvae. Absent Klf9, the diurnal oscillation in *fkbp5* activity was conserved, but *fkbp5* activity was elevated on average (Figure 3A, *p* = 0.007, paired *t*-test), and sinusoid models fit to both WT and *klf9*^–/–^ time-course data indicated a significant elevation of the *fkbp5* MESOR in the mutants (Figure 3B and Supplementary Figure 5). In addition, the circa-dawn peak of *fkbp5* activity was delayed by ∼35 min in *klf9*^–/–^ larvae, occurring ∼30 min before lights-on in WT but several minutes after lights-on in mutants, and the time from peak to nadir was subsequently compressed from ∼3 h in WT to ∼2 h in mutants (Supplementary Figure 6). Combining chronic CORT treatment with the *klf9*^–/–^ mutation cumulatively increased *fkbp5* transcript levels, which were further increased by inhibiting Fkbp5 activity with FK506 (as expected due to loss of Fkbp5-mediated inhibition of the GR), although the magnitude of the latter effect was lower in *klf9*^–/–^ larvae (Supplementary Figure 7).

The qRT-PCR data described above suggested that Klf9 negatively regulates *fkbp5*. To determine if Klf9 physically interacts with *fkbp5*, we performed ChIP-qPCR using primers encompassing putative Klf9 binding motifs identified via JASPAR in the *fkbp5* promoter region (Figure 3C and Supplementary Figure 8). A commercial anti-Klf9 antibody recovered significantly more *fkbp5* promoter region DNA than did a non-specific IgG, and this signal was reduced in *klf9*^–/–^ mutants (Supplementary Figure 9). However, the commercial antibody recognizes an amino acid sequence that is conserved in zebrafish Klf13. We therefore constructed a new zebrafish CRISPR line in which a C-terminal AM epitope tag was introduced into the endogenous *klf9* locus (Supplementary Figure 10 and Supplementary Methods), allowing us to perform Klf9-specific ChIP with the anti-AM antibody. This antibody produced a *fkbp5* ChIP-qPCR signal specific to chromatin from the AM-tagged line (Figure 3D). Together these data indicate that Klf9 interacts physically with the *fkbp5* promoter region.

Because our previous gene ontology analysis suggested that Klf9 regulates steroid metabolism (Gans et al., 2020), we also asked whether the elevation of *fkbp5* transcripts observed in *klf9*^–/–^ larvae might be due to mutants having higher endogenous cortisol levels. We did not, however, detect any difference in either baseline cortisol concentration or diurnal variation in 5 dpf *klf9*^–/–^ larvae, and in fact the cortisol response to an acute stressor was if anything decreased in mutants (Supplementary Figure 4). Thus, the elevated *fkbp5* activity in *klf9*^–/–^ larvae is unlikely to be due to systemically elevated cortisol.

While Klf9 has been implicated in both transcriptional activation and repression in different contexts (Mitchell and Dimario, 2010), it functions predominantly as a repressor in mouse hippocampus, where it binds promoter-proximal DNA enriched for circadian E-Box motifs (Knoedler et al., 2017). HOMER motif enrichment analysis of 149 genes consistently upregulated by chronic CORT (Gans et al., 2020) revealed that 86% of those genes had promoter-proximal KLF and circadian E-Box motifs (while only 46% had glucocorticoid response elements, Supplementary Figure 11 and Supplementary Table 2), with *fkbp5* having E-boxes at +154 and −1,840 bp. As the N-terminal domain of mammalian Klf9 interacts with the Sin3a histone de-acetylation complex (Zhang et al., 2001), we hypothesized Klf9 might repress *fkbp5* via de-acetylation of histone H3 at the *fkbp5* promoter. Using an antibody to H3K14ac and ChIP-qPCR, we found a ∼threefold increase in *fkbp5* promoter DNA recovery in *klf9*^–/–^ larvae compared with WT (*p* < 0.05, Figure 3E). This provides additional evidence that Klf9 directly represses *fkbp5*, forming a circuit predicted to regulate GR activity dynamics through a combination of Fkbp5-mediated negative feedback and Klf9-mediated incoherent feed-forward regulatory logic (Figure 3F).

### Loss of *klf9* Decreases Oxygen Consumption Rate and Alters Metabolic Gene Expression

**FIGURE 4:**
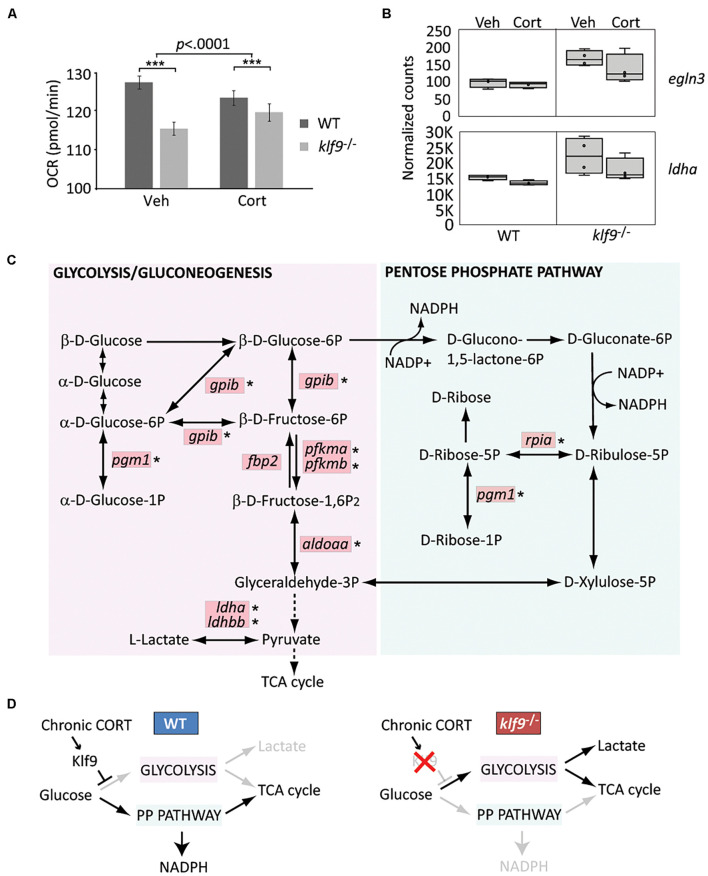
*Klf9* regulates metabolism. **(A)** Oxygen consumption rate (OCR) at 1 dpf, as measured by Seahorse XF96 extracellular flux analyzer. The averages of three experimental replicates are shown, each with 20 embryos per condition measured individually and simultaneously over the course of at least 85 min (thus *n* = 60 total for each condition). The bars represent the average Y-intercept of linear fits of the averaged time-course data (see Supplementary Figure 12), ±standard error. Two-way ANOVA indicated significant effects of both genotype (WT vs. *klf9*^–/–^, *p* < 0.0001) and treatment (VEH vs. CORT, *p* = 0.04), as well as a significant interaction (*p* < 0.0001). ***Adjusted *p* < 0.0001 by Šídák’s multiple comparisons test. **(B)** Normalized counts of Hif1a target *egln3* and glycolytic gene *ldha* (from Gans et al., 2020). **(C)** Krüppel-like factor 9 (Klf9) downregulates multiple genes in the glycolysis/gluconeogenesis pathway. Red boxes indicate genes significantly over expressed (FDR < 0.05) in RNA-seq of *klf9*^–/–^ larvae compared to wild type (Gans et al., 2020). Asterisks denote genes in which putative Klf9 binding sites were identified using HOMER. **(D)** Model for Klf9-mediated regulation of metabolic flux. In response to stress, Klf9 is predicted to inhibit glycolysis, shunting flux through the Pentose Phosphate Pathway (PPP).

The GR, Fkbp5, and Klf9 have all been previously implicated in the regulation of metabolism. To ask if loss of Klf9 affects metabolism in developing zebrafish, we first measured the OCR of 1 dpf embryos and found a 10% decrease in *klf9*^–/–^ mutants compared to WT (Figure 4A and Supplementary Figure 12). We also found a significant interaction between genotype and chronic CORT treatment, which decreased OCR in WT but not mutant larvae, although OCR was still significantly decreased in the mutants compared to WT under chronic CORT (Figure 4A and Supplementary Figure 12).

For additional insight we interrogated our published RNA-seq dataset comparing WT and *klf9*^–/–^ larvae (Gans et al., 2020) by performing a focused principal component analysis (PCA) on two gene lists (Supplementary Table 3) comprising targets of Hif1 and AMPK signaling, which respectively regulate glycolysis and oxidative metabolism. These gene lists were previously shown to accurately reflect changes in metabolic phenotype of cancer cells, which rely more heavily on glycolysis as they become more malignant (i.e., the Warburg Effect) (Jia et al., 2019). The first principal components (PC) of both datasets (respectively, accounting for 28 and 27% of the variance) represented the effect of chronic CORT treatment, and plotting them against each other reveals correlation between the two pathways (Supplementary Figure 13A). Top genes contributing to AMPK PC1 included insulin regulator *mafa* at the VEH pole, while the CORT-treated pole was weighted by the GC target *g6pca*, two members of the stress-responsive *gadd45* family, and *acadm*, which encodes a catalyst of mitochondrial beta-oxidation. Klf9^–/–^ mutant samples showed a shift away from the CORT pole of AMPK PC1, suggesting less influence of CORT on expression of these genes in mutants. Plotting PC1 vs. PC2 within either the Hif1 or AMPK dataset segregated the samples by treatment and genotype (Supplementary Figure 13B). Hif1 PC2 (12% of variance) segregated WT from *klf9*^–/–^ mutant samples; examples of genes at either end of this axis included the Hif1a regulator *egln3*, upregulated in mutants (Figure 4B), and *eprs1*, downregulated in mutants. Lactate dehydrogenase (*ldha*), a noted Hif1 target (Lee et al., 2015; Cui et al., 2017), was also upregulated in *klf9*^–/–^ mutants (Figure 4B). Wild-type samples spanned AMPK PC2 (19% of variance), whereas *klf9*^–/–^ samples were restricted to one pole (Supplementary Figure 13B), indicating less variation in expression in mutants of these genes, which included *pck1* and *pck2*.

We previously reported that genes involved in glycolysis and pyruvate metabolism were reciprocally regulated in *klf9*^–/–^ and GR^–/–^ mutants, being upregulated in the former and downregulated in the latter (Gans et al., 2020). KEGG pathway analysis of the full set of genes significantly affected in *klf9*^–/–^ larvae under all conditions (FDR *q* < 0.05, Supplementary Table 4) showed enrichment for genes involved in glycolysis/gluconeogenesis as well as the adjacent pentose phosphate pathway (PPP) (Figure 4C, FDR < 0.01). We further parsed this by separate Gene Ontology comparisons between WT and *klf9*^–/–^ larvae in VEH or chronic CORT conditions. Processes up-regulated in CORT-treated *klf9*^–/–^ vs. CORT-treated WT larvae again included glycolysis and pyruvate metabolism (Supplementary Figure 14 and Supplementary Table 4), while processes upregulated in mutants under VEH conditions were instead largely involved in innate immunity as reported previously (Gans et al., 2020). These data suggest that chronic CORT exposure activates a metabolic stress response that unmasks the role of Klf9 in regulating genes involved in glycolysis and related processes. We used HOMER to look for enriched transcription factor binding motifs within 2,000 bp of the transcription start site (TSS) of genes upregulated in mutants treated with CORT. The consensus motif for Klf9 was the highest scoring motif on the list (Supplementary Figure 14), and seven of the top ten were highly similar binding motifs for other KLF factors that Klf9 could also be expected to bind (Ilsley et al., 2017). Notably, except for *fbp2*, all the glycolytic and PPP genes overexpressed in *klf9*^–/–^ mutants had potential KLF sites (Figure 4C). In contrast, genes downregulated in mutants showed no enrichment for KLF motifs, and were instead enriched for motifs for IRFs, HNF4a, CREB5, and the retinoic acid receptor. These data suggest that Klf9 functions predominantly as a repressor as previously reported (Knoedler et al., 2017), leading us to hypothesize that it regulates metabolism in part by repressing glycolytic genes, with a predicted effect of shunting flux through the PPP (Figure 4D). Further studies are required to test this hypothesis, using ChIP to assay Klf9 binding to putative KLF sites in metabolic genes, and analysis of metabolites to test predicted effects of *klf9* mutation.

## Discussion

We have shown that in zebrafish the GC-responsive genes *klf9* and *fkbp5* are expressed with synchronous oscillatory dynamics that are distinct from those of other GC-responsive genes we examined. *FKBP5* is a gene of high biomedical interest given its role in GR regulation, maladaptation to stress, and mental and metabolic health (Hausl et al., 2019; Zimmer et al., 2020). *Klf9* has also been linked to a maladaptive stress response (Besnard et al., 2018), and our results suggest that the GR, *klf9*, and *fkbp5* comprise a “hardwired” genetic circuit. *FKBP5* was previously reported to be repressed by KLF9 in human epidermis (Sporl et al., 2012), and we show here that loss of Klf9 function in zebrafish leads to elevated *fkbp5* transcript levels and hyperacetylation of chromatin at the *fkbp5* promoter, and that Klf9 physically associates with that chromatin, supporting that *fkbp5* is a target of Klf9-mediated repression. The proposed GR-*klf9-fkbp5* regulatory circuit (Figure 3F) combines negative feedback and incoherent feedforward loops, motifs predicted to facilitate adaptation to repeated or chronic stimuli (Ma et al., 2009; Shi et al., 2017; Alon, 2019). Further study is necessary to test this hypothesis and explore its ramifications for specific cell and tissue types under different conditions affecting GC signaling, and to determine how the transcript dynamics reported here relate to protein dynamics.

The evidence reported here and in our previous study (Gans et al., 2020) that *klf9* both responds to and regulates GC signaling, together with our observation that *klf9*^–/–^ mutants have reduced OCR at 1 dpf and increased expression of glycolytic genes at 5 dpf, suggests that *klf9* is instrumental in regulating metabolism and metabolic responses to cortisol (Denver et al., 1999; Bagamasbad et al., 2015; Pabona et al., 2015). Repression of glycolytic genes by Klf9 fits with its reported inhibition of growth and regeneration (Apara et al., 2017; Galvao et al., 2018) and regulation of stem-cell metabolism (Cvoro et al., 2015) and could also contribute to its role in tumor suppression (Sun et al., 2014; Zhong et al., 2018), given the reliance of cancer cells on glycolysis. Shunting flux through the PPP is predicted to increase NADPH, which fits with the known role of Klf9 as a regulator of redox homeostasis (Zucker et al., 2014). It is important to emphasize, however, that we do not yet know the mechanisms underlying the effects of either the *klf9*^–/–^ mutation or chronic CORT on the OCR in 1 dpf embryos. A previous study reported that morpholino knockdown of *klf9* disrupts erythropoiesis in zebrafish (Zhang et al., 2017), suggesting decreased OCR in *klf9*^–/–^ larvae could result from a deficit in red blood cells. However, in that study primitive hematopoiesis was unaffected, and no differences observed prior to 2 dpf (whereas we measured OCR at 1 dpf), which makes that explanation unlikely. While our gene expression results suggest that loss of *klf9* function produces a metabolic shift toward glycolysis, those results were obtained in 5 dpf larvae, and may not be directly relatable to the differences in OCR observed at 1 dpf, given the significant development that occurs between 1 and 5 dpf. Our results are nonetheless consistent with evidence of cell autonomous metabolic regulation in mice, where over-expression of *klf9* increased oxygen consumption and the number of mitochondria in cultured adipocytes (Fan et al., 2020). Similarly, elimination of *fkbp5* elevates resting metabolic rate while protecting against obesity and increasing glucose tolerance (Balsevich et al., 2017). *FKBP5* expression and blood glucose/insulin resistance are positively correlated in humans (Sidibeh et al., 2018), and a mutation causing elevated *FKBP5* is associated with reduced weight loss following bariatric surgery (Pena et al., 2020).

Induction of hepatic *klf9* by dexamethasone promotes gluconeogenesis and hyperglycemia via PPAR activation, while *klf9* knockout animals display hypoglycemia (Cui et al., 2019). PPAR promotes PPP flux as well as *de novo* lipogenesis (Summermatter et al., 2010). Interestingly, we found that promoters of genes under-expressed in *klf9*^–/–^ mutants are enriched for sequence motifs recognized by the retinoic acid receptor, known to dimerize with PPAR (Keller et al., 1993). Lipid signaling may be an indirect means by which Klf9 regulates gene expression, which may account for the enrichment for IRF motifs among genes upregulated by CORT treatment in WT but not *klf9*^–/–^ larvae (Gans et al., 2020) as lipid signaling regulates interferon levels (Hara et al., 2003; Den Brok et al., 2018). The Klf9-dependency of GC-induced immune gene expression (Gans et al., 2020) may be secondary to metabolic differences in *klf9*^–/–^ mutants, as reactive oxygen species, disruption of glycolytic flux, and the accompanying drop in NADH promote inflammasome activation (Sanman et al., 2016; Yang et al., 2019). Further studies are required to test these possibilities.

## Data Availability Statement

The datasets presented in this study can be found in online repositories. The names of the repository/repositories and accession number(s) can be found below: https://www.ncbi.nlm.nih.gov/, GSE144885.

## Ethics Statement

The animal study was reviewed and approved by the Institutional Animal Use and Care Committee, MDI Biological Laboratory.

## Author Contributions

IG and JC: conceptualization, writing—original draft, and visualization. IG, RB, NJ, and JC: methodology and writing—review and editing. IG, JG, and RB: investigation. NJ: resources. JC and NJ: supervision. JC: project administration and funding acquisition. All authors contributed to the article and approved the submitted version.

## Conflict of Interest

The authors declare that the research was conducted in the absence of any commercial or financial relationships that could be construed as a potential conflict of interest.

## Publisher’s Note

All claims expressed in this article are solely those of the authors and do not necessarily represent those of their affiliated organizations, or those of the publisher, the editors and the reviewers. Any product that may be evaluated in this article, or claim that may be made by its manufacturer, is not guaranteed or endorsed by the publisher.
