# Supplementary material for: Glucocorticoid-Responsive Transcription Factor Krüppel-Like Factor 9 Regulates fkbp5 and Metabolism
Source: Front Cell Dev Biol. 2021 Oct 6;9:727037. doi: 10.3389/fcell.2021.727037 (PMC8526736; doi:10.3389/fcell.2021.727037)
Supplement: Supplementary file 1 [file Data_Sheet_1.PDF]

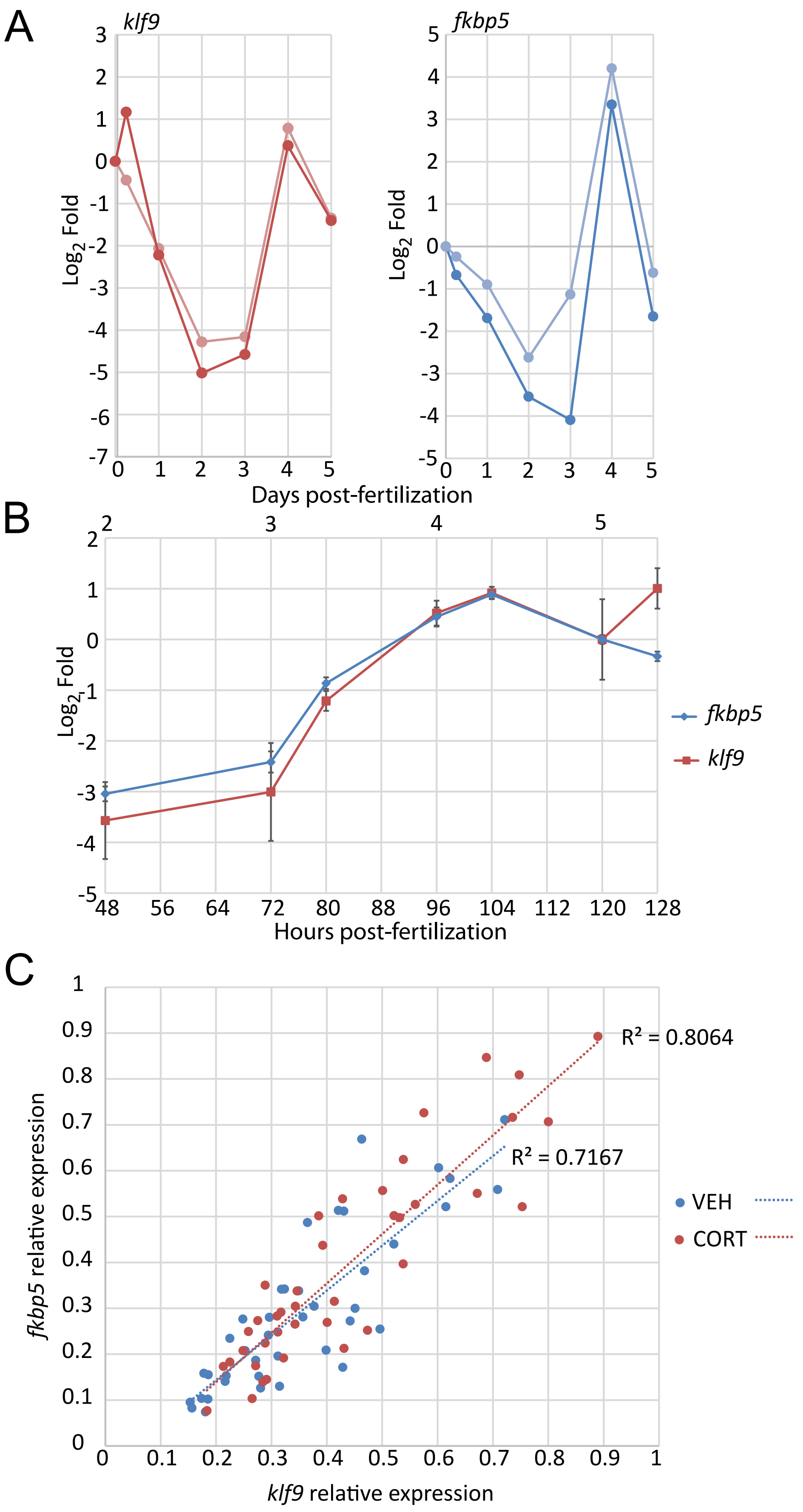

Figure S1. Expression of *klf9* and *fkbp5* is highly correlated. (A) Relative mRNA levels of each gene measured by qRT-PCR in two biological replicates over the first five days of development, showing that transcripts of both genes are maternally deposited and substantially depleted by 2dpf, and that expression levels rise again after 3dpf, following development of the larval HPI axis. (B) Relative mRNA levels of each gene in a third biological replicate measured by qRT-PCR on days 2-5 postfertilization. (C) Correlation of data points from the 5 dpf time-courses shown in Figures 1 and 2.

A

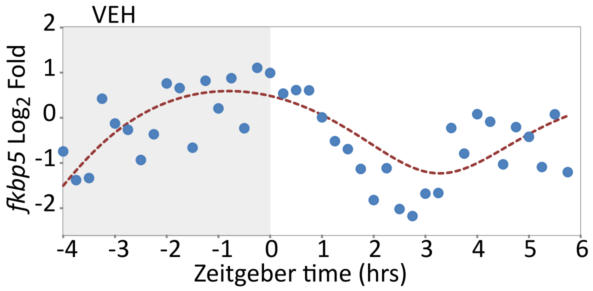

Source	Degrees freedom	Sum of squares	Mean square	F	P value
Model	4	5.56	1.39	9.03	4.03E-05
Error	35	5.38	0.15		
Total	39	10.9			

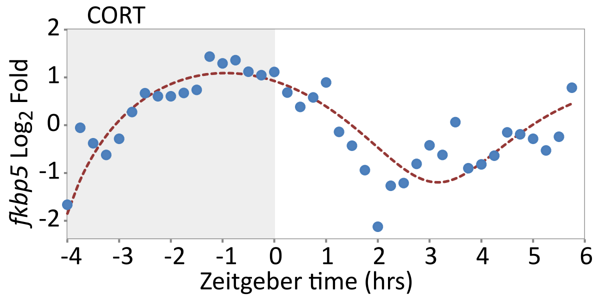

Source	Degrees freedom	Sum of squares	Mean square	F	P value
Model	4	1.46	0.364	30.5	5.73E-11
Error	35	0.418	0.012		
Total	39	1.87			

B

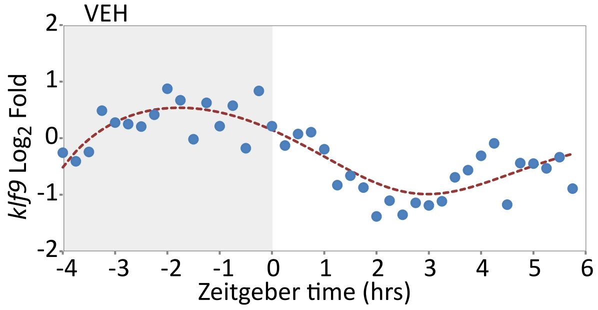

Source	Degrees freedom	Sum of squares	Mean square	F	P value
Model	4	4.34	1.09	22.7	2.54E-09
Error	35	1.67	0.05		
Total	39	6.01			

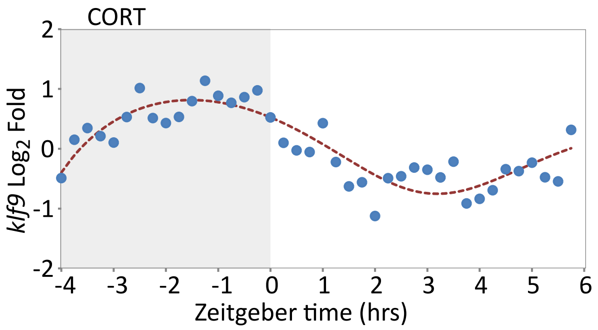

Source	Degrees freedom	Sum of squares	Mean square	F	P value
Model	4	6.57	1.64	30.5	5.60E-11
Error	35	1.88	0.05		
Total	39	8.45			

Figure S2. Temporal variation of (A) *fkpb5* and (B) *klf9* mRNA levels in 5 dpf larvae fit to sinusoidal models (dashed lines). Each data point is the average of three experiments (also shown in Figure 2). Tables on the right show ANOVAs and calculated P-values for the fit of the data to the model.

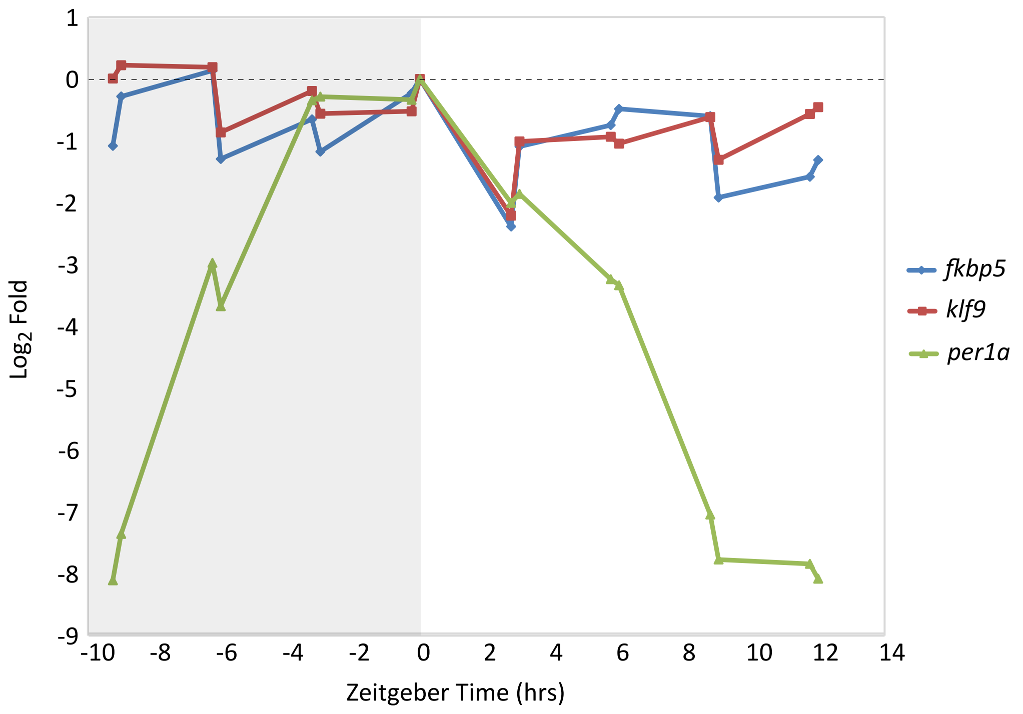

Figure S3. Circadian variation of *fkbp5*, *klf9*, and *per1a* mRNA levels in 4-5 dpf larvae.

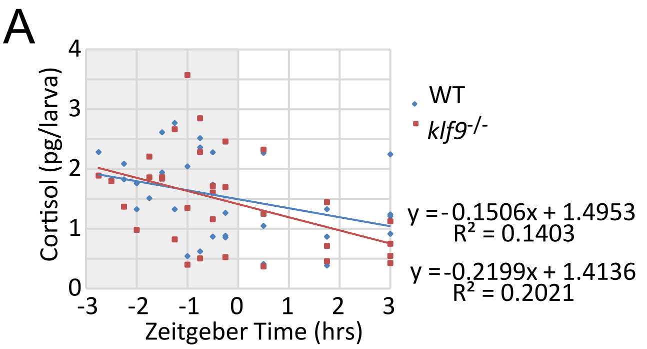

Linear Regression Model
Coefficients:

	Estimate	Std. Error	t value	Pr(>|t|)	
(Intercept)	1.45379	0.08708	16.69	< 2e-16	***
ZT	-0.18469	0.05173	-3.57	0.000696	***

Residual standard error: 0.689 on 62 degrees of freedom
Multiple R-squared: 0.1705, Adjusted R-squared: 0.1572
F-statistic: 12.75 on 1 and 62 DF, p-value: 0.0006957

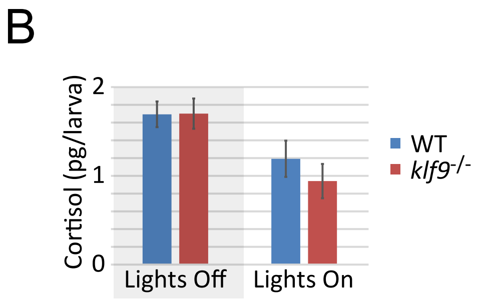

2-WAY ANOVA RESULTS

	Df	Sum Sq	Mean Sq	F value	Pr(>F)	
geno	1	0.085	0.085	0.173	0.67924	
lights	1	5.468	5.468	11.048	0.00152	**
geno:lights	1	0.231	0.231	0.466	0.49748	
Residuals	60	29.695	0.495			

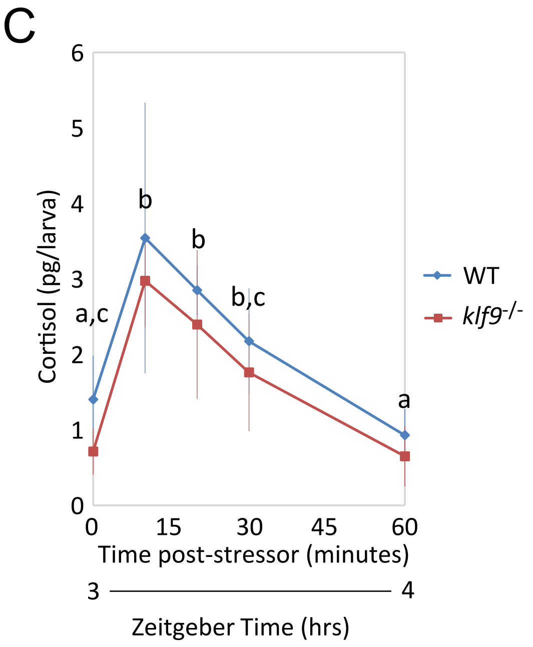

Figure S4. Cortisol levels in pooled 5 dpf larvae measured by ELISA. (A) Linear regression shows a significant effect of zeitgeber time (ZT) on baseline (non-stressed) whole body cortisol ($p<0.001$). Each data point represents a single sample of pooled larvae (n=15-20 larvae per sample). (B) Re-analysis of data from (A) binned categorically and analyzed by two-factor ANOVA (genotype x lights/time) shows a significant effect of lights/time on cortisol ($p=0.00152$). Error bars represent standard error of the mean. (C) Cortisol response to acute pipette chase stress. Data points are averages of four biological replicates of pooled larvae (n=10-20 larvae per sample). Two-factor ANOVA indicated a significant effect of time post-stressor on cortisol ($p < 0.0001$), and a trend ($p=0.07$) toward lower cortisol in mutants. Letters indicate significant differences ($p < 0.05$) among time points according to Tukey's post-hoc test. Error bars represent the standard deviation.

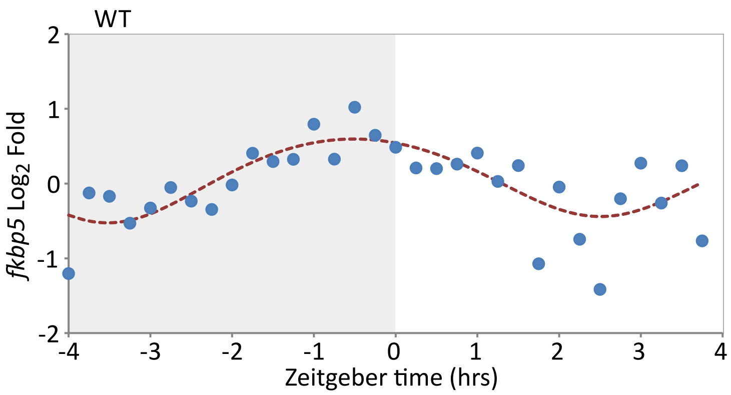

Source	Degrees freedom	Sum of squares	Mean Square	F	P value
Model	4	2.53	0.633	10.2	3.70E-05
Error	27	1.68	0.062		
Total	31	4.21			

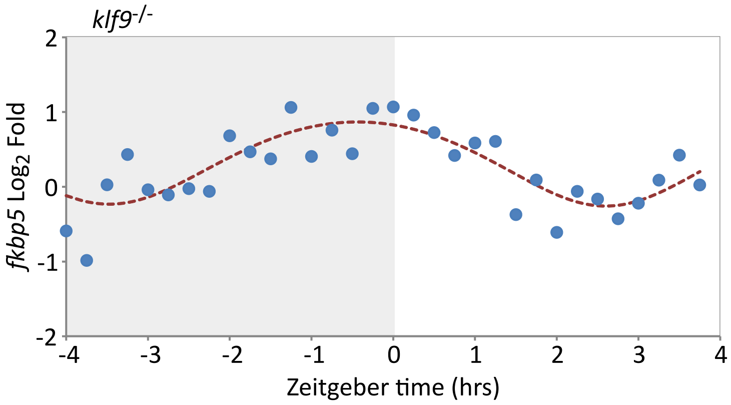

Source	Degrees freedom	Sum of squares	Mean Square	F	P value
Model	4	3.91	0.977	13.4	3.88E-06
Error	27	1.97	0.073		
Total	31	5.88			

Figure S5. Temporal variation of *fkbp5* mRNA levels in 5 dpf wildtype (WT) and *klf9*$^{-/-}$ larvae fit to sinusoidal models. Each data point is the average of three experiments in the indicated genotypes (also shown in Figure 3). Tables on the right show ANOVAs and calculated P-values for the fit of the data to the model.

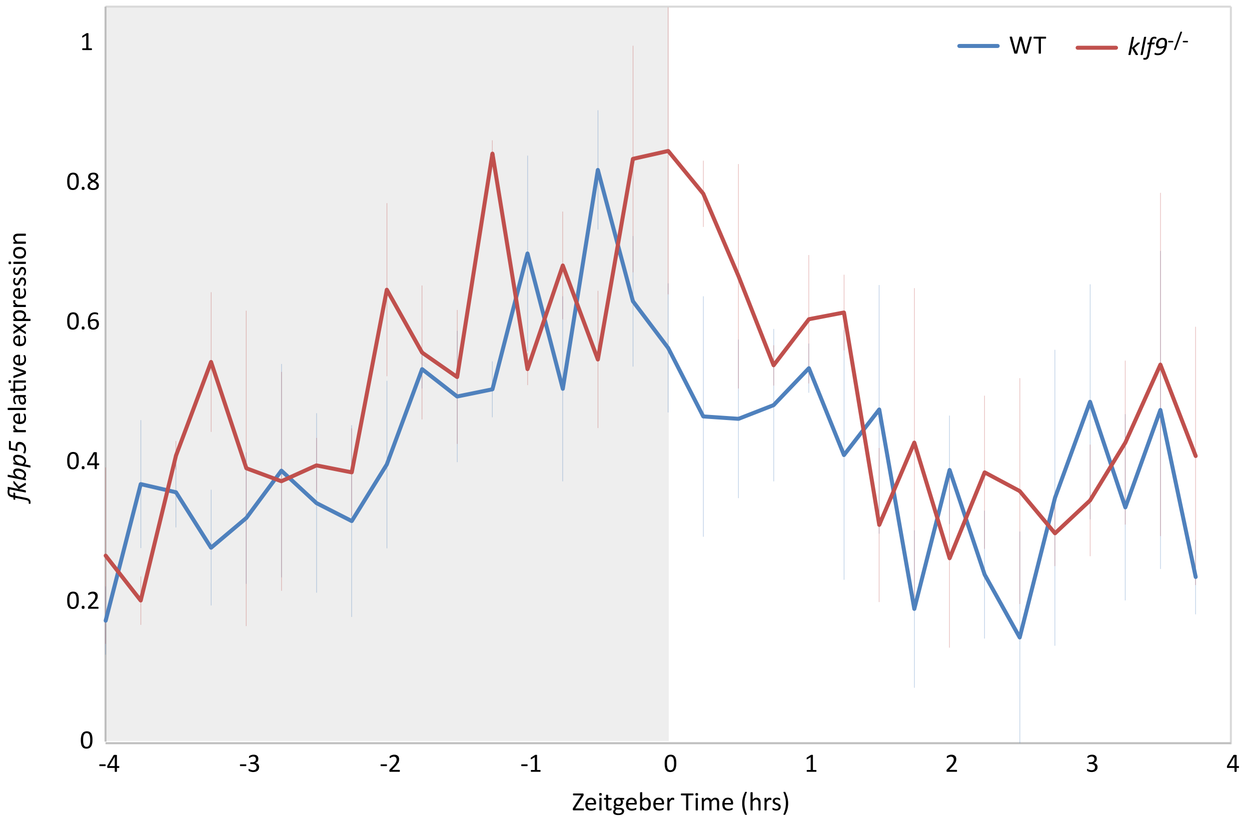

Figure S6. Temporal variation of *fkbp5* mRNA levels in 5 dpf wildtype and *klf9*$^{-/-}$ larvae (same data as shown in Figure 3A, replotted as a line graph). Note the delayed peak expression in *klf9*$^{-/-}$ mutants at ~0 ZT. Error bars represent standard error of the mean of three biological replicates.

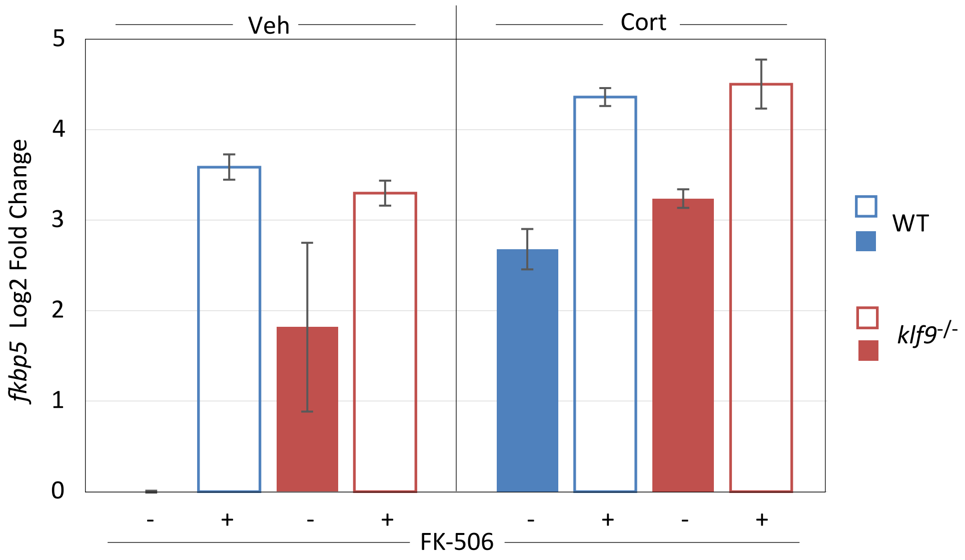

Figure S7. Relative expression of *fkbp5* in 5 dpf WT and *klf9*$^{-/-}$ larvae treated with VEH or chronic 1uM CORT from 0-5dpf, and FK-506 or additional vehicle control overnight from 4-5dpf. Error bars represent the 95% confidence interval of two biological replicates.

GR: LASAGNA Transfac M00205; **KLF9**: JASPAR MA1107.1

>*Dr_fkbp5*_upstream3000-exon1_refGene_NM_213149

ctcttacacaaaacccatctgttatgtca**ctcacattatgttc**ctggattaactatccatctgaaattattttaaa
aatcaggcttaaaaaagggaaatcaatcccttcaaacgcaggcaatcacgtatgccacctattgtgttcctgctgac
acctcttgttgctgtggtgaacgagccg**ggaacattttgtaac**agtttcagcctggagaggcttttgaggcaggtaa
catgttgccattactgatgttattcaatgtttggcatggtaacttgatagactggatttgatcagaggatgatcacg
atgtacaatgctttcatgtagacattgtaaacatgaggttatttataattatttattgaaaatgttacttagcttta
aagattcattgtggattaaacttaacttttaattaggcaatattcgcccccacttcagggacaatataaagtcaat
aaatcaaatactaattggatcaatgttgccttttaaggcaaatacctaaattgaatctattacttcagtcaaccaca
aataacaacaacatgttaaaagttgtacttcaattatccacttaaccaattaactcattactgggtaatgctgtggt
cacactaaagttttagatgcaaaattgtgtccatattttaaaatttctgtacagagaggtcatgttttgattttcg
attagttttgcgaagtcaagtgatgcaatttcacaggtcagagttcaccaagcttgaaccttcaaatgcagtgaact
acgaaacttgtcgcacaagcttgcatttccagtttgttgctttcgcatgtgattgaatggaagtctaaggggcaaaa
tgagcagtgtgaccatggcttttgctggtaaatatattgattaattaacagttgctcatgatttatggatgttctcc
aattatacagttttaaattgcattatagaatcttaattattgctccaaccaattctgatatttaaaaaataactctg
caggttaaaaaactgactgaaataggcctttcaatagtttgaaataatttattgtataacatgtaatacatatata
acagaaaaagtactgaaagattaacaaatggtcttgattacctagacagtatatattgatggattgattgaacttca
catgataaagctgcaaaaatgtctatctatctatctatctatctatctatctatctatctatctatctatctatcta
tctatctatctatctatctatctatctatctatctatatctatctatctatctatctatctatctatctatctatct
atctatctatctatatatctaactatccatccatccatccatccatccatccatccatccatccaccca**tatacaca**
cacacacacacacacacacatatttatatatatatatattatgcacatacatacatacatacatacatacatatc
agggctgtgtgatttggagaaaatatcaaattaaatttttttgacaaatattgttattgcgatttgaattacggttt
aattttgaagcagcagacgccataatcccacttgtcaatgctttcctttttttattgtaggcataattcagtagcgc
aattctgaaatggcagaatgaaagtgtgcccactcagttggctagttataaagccctggctggatcacacgatttcg
acatgatttccttcacgttgggtgtcgtggggagttgtaaatgactccaatagcatctggatgcagcctttatatgc
aagctgagattgcatcactcagcgatgcaatgtcagacaaaatgcactcaatggagtgagtgacgtcactgtacagc
gacaaatggatgtcatgacacaaaatttttcttctcgtgtaatgtgccatagttt**acgacagctgatacc**atgtct
catgacaccatcgcatgtttaatttcggactggttttgaagcacctcagatgctattcctcatcctggtttagata
aatacatattattaaatatatatagaggcctatatata**tgtgtgtgtgtgtgtgtgtgtgtgtgtgtgtgtgtgtgt**
gtatgtatgtatgtatatatatatatatatatatatatatatatatacacataatatatacataactaattgcaaca
tttcaagttgcgattgcgatttgactttgattaatcgcagagcttgttaattaatgatctctttatttttgtaacaa
gatctgcatcaaatatatggaagaaaaattattagcataatacttattgaaaaatctacaacaagcattaaacaaat
tagtgaacttatatttaaaaactatgctttatcgtatgaaaatacaggtttctttacaaactacagtaaaaaaaagc
ttctgcgcgttttataactttgaataataataataataataataataataatcatgatgatgatgttgaattat
atcgctacaccggctctattcgattttatgctcaatatgctgctttagttctaggcttggtatagtgtcatcccgat
ggtgttg**tgtgcagtctgttct**tgctttctgtttcatcacctgcatcctcatcagaactgtaaaatataaacagcct
acca**tccaacacccaca**atcgtcttcgtagtatgtttttctctacttatttttttacttccacatttttctcgac
gcaatctagtcgtgttgttctttccgcgtatcgctgtgctctgtgtggt**tgtacacgctgtt**cctaacttgagctgt
tcactcagcacacaacgggttacgggtcagggtgtctact**aggacactgtgttct**catcagcgcgagctac**agtaca**
gccggaacattgtgttactttgtggacagttt**ccaaggcctgcccttaattt**ta**ctccacaccgccc**tcccccagcg
tttactcctcctcctttagtctcatttcatttccccc**gtccaaatgtctcgcagagg**cgtatcgtgttgatgatTAT
TGGTGTGGAGAAGTGTAAAATACTGCCGGT**AGAACATCTAGTACT**CTCGCGGTGCTGAGAGGGCGGGCTCATTCCCC
TTTAAGCCGCTACGCACAGGTTGTATTCGCCGGTGAGACTAAACAGAAGTTGTATACGGACGGATAAATGGTCAAAC
ACATGAGAGGAAACAACAACCAAAGGAATATTTTGGGAACAAGGGCATGTATGAGTGGTGAG

Figure S8. Genomic sequence upstream of *Danio rerio fkbp5* exon 1, highlighting Klf9 binding motifs (red) as well as glucocorticoid receptor (GR) binding motifs (green) identified respectively by JASPAR and LASAGNA using the indicated motif matrices. Upstream sequence (3 kb) is in lower case font; exon 1 sequence is in upper case. Primer target sequences used for ChIP-qPCR are underlined.

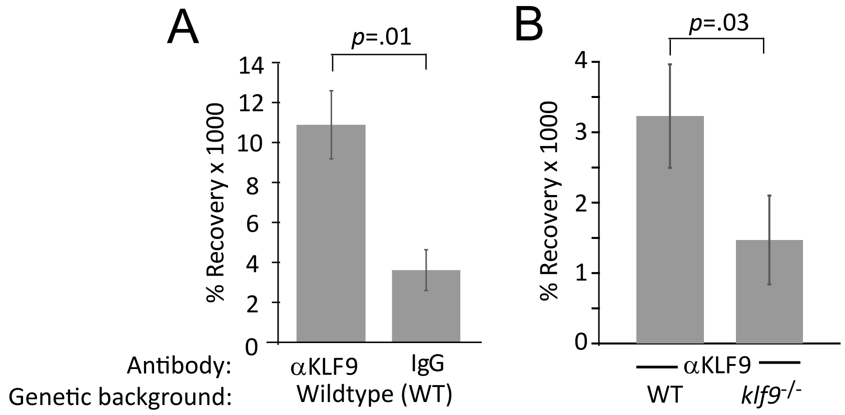

Figure S9. qPCR measurements of *fkbp5* promoter-proximal sequence (see Figure 3C) recovered by ChIP using a commercial anti-Klf9 antibody. (A) Recovery of *fkbp5* DNA from 5 dpf wildtype larvae by ChIP using the anti-Klf9 antibody vs. a nonspecific IgG isotype control. Data are mean of four experimental replicates with standard error. (B) Recovery of *fkbp5* DNA by ChIP using the anti-Klf9 antibody from 5 dpf wildtype vs. *klf9*$^{-/-}$ larvae. Data presented are mean of two experimental replicates with standard error. Significance calculated by Student's paired T-test.

A Donor sequence with C-terminal AM tag flanked by homology arms

AGACGACACTCTTCAACATCCACATCCTCCTCTGGC*TCCAGCGATCAAATGAGCGC*T
GGTGTTTGCCAAGATCCTCAACGCAAAGGCAACGTGATACTCTCTCAGGCTTACGGG
TGCCAAGATCCTCAACGCAAAGGCAACGTGATACTCTCTCAGGCTTACTAAgcattt
tgactgtctct***cctacctgacacaacacgtt***caggagtctctgctagtgagttggtg

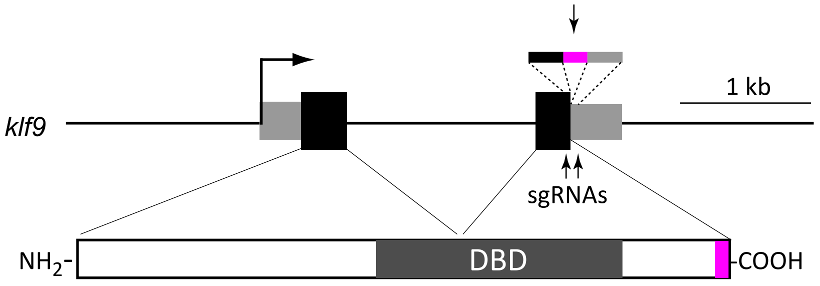

B Sequence of PCR amplicon from F1 fin clip

AGACGACACTCTTCAACATCCACATCCTCCTCTGGCTCCAGCGATCAAATGAGCGCT
GGTGTTTGCCAAGATCCTCAACGCAAAGGCA***ACGTGATACTC****TCTCAGGCTTACGGG*
TGCCAAGATCCTCAACGCAAAGGCAACGTGATACTCTCTCAGGCTTACTAAgcattt
tgactgtctctcctacctgacacaacacgttcaggagtctctgctagtgagttggtg

Figure S10. Construction of AM epitope-tagged Klf9 line. (A) Schematic of donor template and strategy for CRISPR-mediated insertion of a C-terminal AM tag into the *klf9* locus by homology directed repair. AM (Active Motif) sequence shown in magenta is flanked by microhomology arms of endogenous exonic (black) and intronic (grey) sequence; sgRNA target sites are italicized. Silent and PAM mutations (red) were included to prevent re-editing. A schematic of the resulting protein shows the location of the resulting epitope tag (“DBD”, DNA binding domain). (B) Assembled sequence of PCR product amplified from genomic DNA extracted from fin clip of F1 progeny of Klf9-C-term-AM-tag founder x wildtype outcross, with locations of forward and reverse sequencing primers underlined, sequence from forward primer in italics, overlapping sequence in bold font.

A

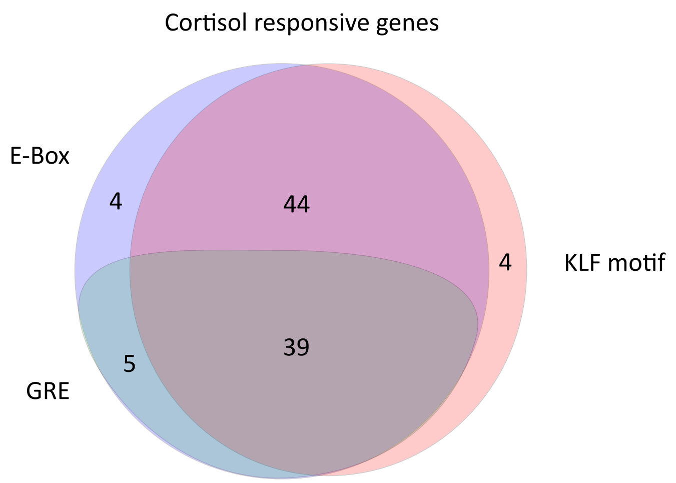

B

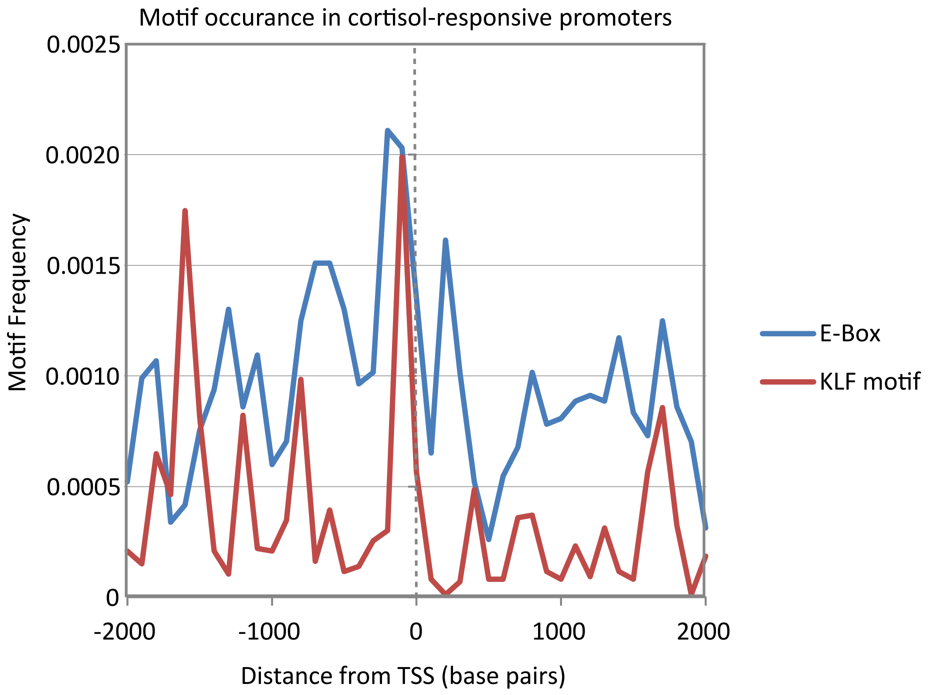

Figure S11. DNA sequence flanking the transcriptional start site (TSS) of cortisol-responsive genes identified by RNA-seq contains a high frequency of KLF binding motifs and E-boxes, as well as glucocorticoid response elements (GREs). (A) Prevalence of E-boxes, KLF binding motifs, and GREs in 96 genes previously identified as being consistently responsive to cortisol treatment (Gans et al., 2020), and their overlap. (B) Frequency distribution of E-boxes and KLF binding motifs with respect to the TSS.

A

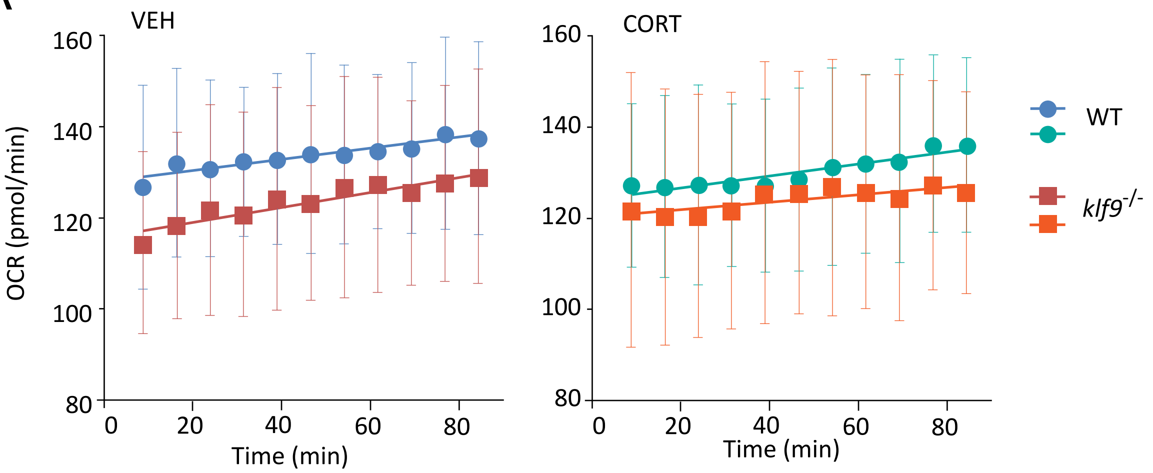

VEH	WT	*klf9*$^{-/-}$
Best-fit values		
Slope	0.1189	0.1659
**Y-intercept**	**128.0**	**115.8**
X-intercept	-1076	-698.1
1/slope	8.410	6.029
Std. Error		
Slope	0.03199	0.03607
**Y-intercept**	**1.673**	**1.886**

CORT	WT	*klf9*$^{-/-}$
Best-fit values		
Slope	0.1340	0.08269
**Y-intercept**	**123.9**	**120.1**
X-intercept	-924.9	-1453
1/slope	7.464	12.09
Std. Error		
Slope	0.03233	0.04309
**Y-intercept**	**1.691**	**2.254**

B

**Table Analyzed**	**Grouped: Two-way ANOVA (two data sets)**
Two-way ANOVA	Ordinary
Alpha	0.05

**Source of Variation**	**% of total variation**	**P value**	**P value summary**	**Significant?**
Interaction	13.32	<0.0001	****	Yes
Row Factor (treatment)	0.1740	0.0416	*	Yes
Column Factor (genotype)	76.72	<0.0001	****	Yes

**ANOVA table**	**SS**	**DF**	**MS**	**F (DFn, DFd)**	**P value**
Interaction	735.0	1	735.0	F (1, 236) = 321.3	P<0.0001
Row Factor	9.600	1	9.600	F (1, 236) = 4.197	P=0.0416
Column Factor	4234	1	4234	F (1, 236) = 1851	P<0.0001
Residual	539.9	236	2.288		

Figure S12. Measurement of oxygen consumption rate with the Seahorse XF96 extracellular flux analyzer. (A) The plots show the averages +/- standard deviations of 60 embryos per indicated genotype/treatment group (3 experimental replicates with 20 embryos each) at each indicated time point. The tables show the linear regression of the data yielding the y-intercept and standard error values plotted in Figure 4A. (B) Two-way ANOVA assessing effects of the *klf9*$^{-/-}$ mutation (column), CORT treatment (row), and their interaction.

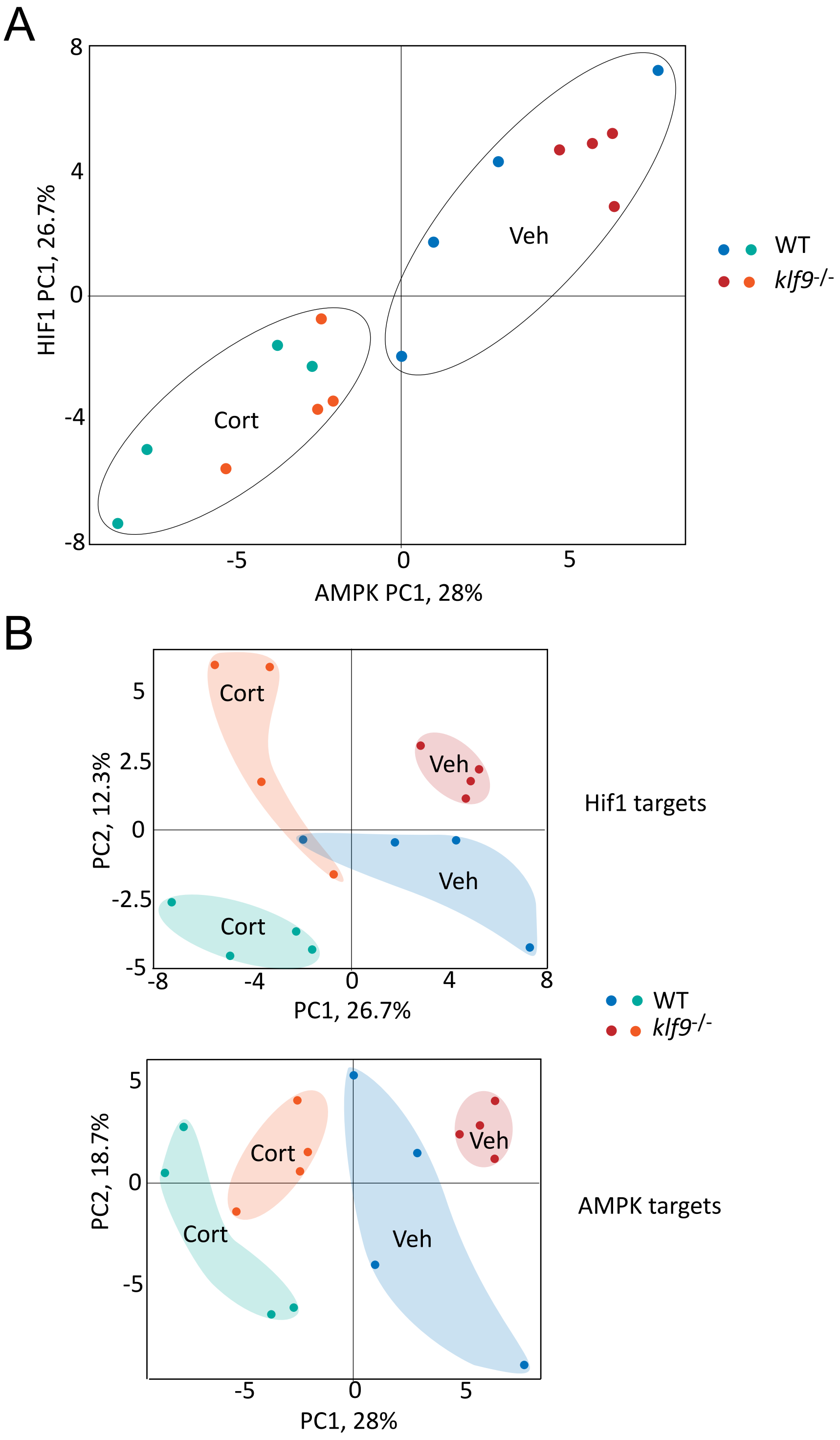

Figure S13. PCA of Hif1 and AMPK pathway target genes (subset of RNA-seq data from Gans et al., 2020). (A) Plotting PC1 of Hif1 target expression versus PC1 of AMPK target expression segregates samples by treatment and indicates strong co-regulation of the two pathways. (B) Plots of principal components 1 and 2 for genes in each pathway.

A

REVIGO Gene Ontology treemap

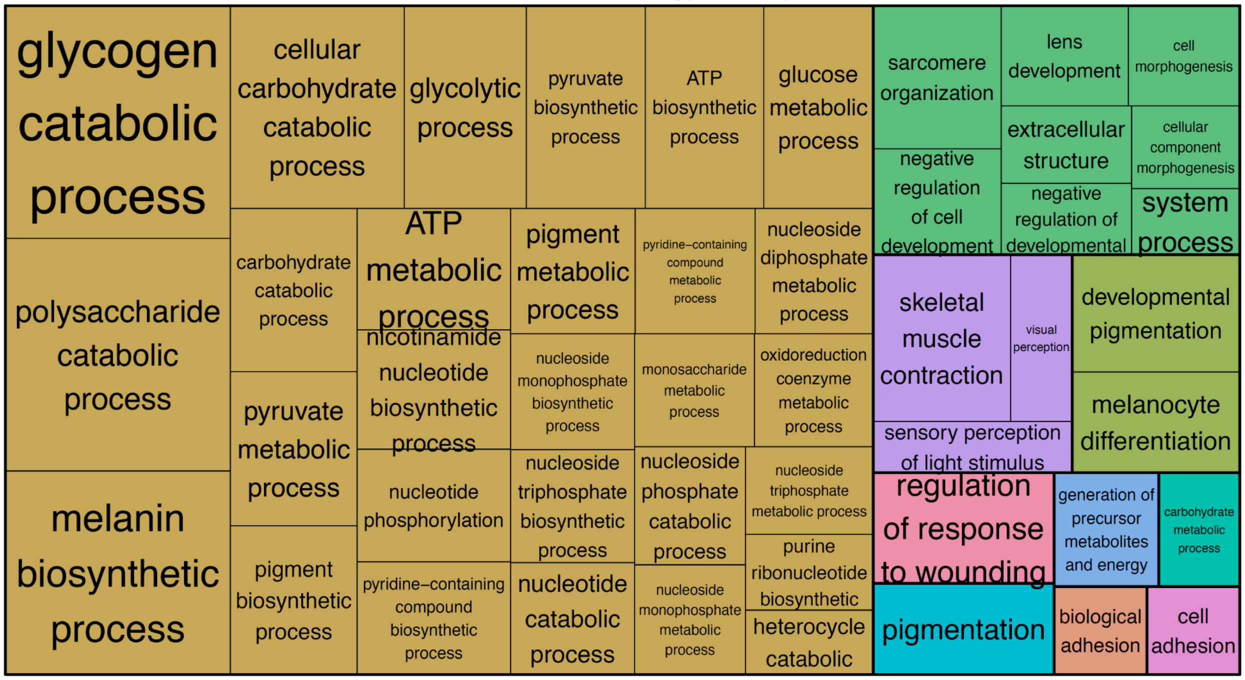

B

Rank	Motif	Name	P-value	log P-pvalue	q-value (Benjamini)
1	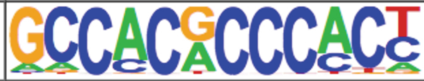	Klf9(Zf)/GBM-Klf9-ChIP-Seq(GSE62211)/Homer	1e-7	-1.646e+01	0.0000
2	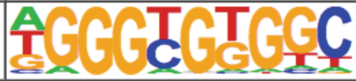	KLF5(Zf)/LoVo-KLF5-ChIP-Seq(GSE49402)/Homer	1e-5	-1.305e+01	0.0005
3	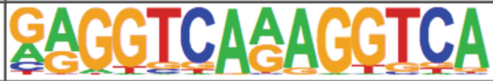	TR4(NR),DR1/Hela-TR4-ChIP-Seq(GSE24685)/Homer	1e-5	-1.299e+01	0.0005
4	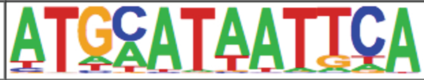	Pit1+1bp(Homeobox)/GCrat-Pit1-ChIP-Seq(GSE58009)/Homer	1e-5	-1.292e+01	0.0005
5	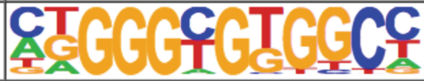	KLF6(Zf)/PDAC-KLF6-ChIP-Seq(GSE64557)/Homer	1e-5	-1.264e+01	0.0005
6	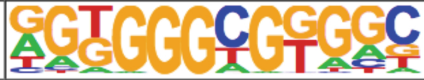	KLF14(Zf)/HEK293-KLF14.GFP-ChIP-Seq(GSE58341)/Homer	1e-5	-1.157e+01	0.0007
7	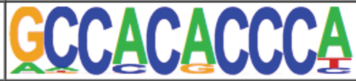	Klf4(Zf)/mES-Klf4-ChIP-Seq(GSE11431)/Homer	1e-4	-1.084e+01	0.0012
8	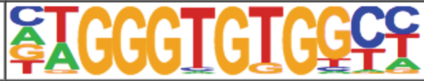	EKLF(Zf)/Erythrocyte-Klf1-ChIP-Seq(GSE20478)/Homer	1e-4	-9.962e+00	0.0026
9	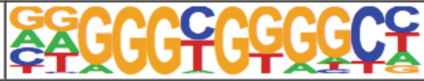	KLF1(Zf)/HUDEP2-KLF1-CutnRun(GSE136251)/Homer	1e-3	-8.150e+00	0.0141
10	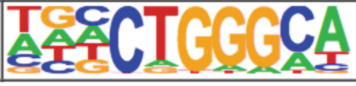	ZNF416(Zf)/HEK293-ZNF416.GFP-ChIP-Seq(GSE58341)/Homer	1e-3	-7.996e+00	0.0148

Figure S14. Loss of Klf9 function leads to upregulation of metabolic genes, and sequences near the transcriptional start sites (TSS) of those genes are enriched for Klf9 sites. (A) REVIGO tree map of processes upregulated by chronic CORT treatment in *klf9*$^{-/-}$ mutants compared to CORT-treated WT larvae. (B) The top ten most significantly enriched motifs identified by HOMER within +/- 2000bp of TSS of genes depicted in (A).
