# Supplementary material for: Glucocorticoid-Responsive Transcription Factor Krüppel-Like Factor 9 Regulates fkbp5 and Metabolism
Source: Front Cell Dev Biol. 2021 Oct 6;9:727037. doi: 10.3389/fcell.2021.727037 (PMC8526736; doi:10.3389/fcell.2021.727037)
Supplement: Supplementary file 2 [file Data_Sheet_2.PDF]

Glucocorticoid-responsive transcription factor Klf9 regulates *fkbp5* and metabolism

Ian M. Gans[1,2], Janelle Grendler[1], Remy Babich[3], Nishad Jayasundara[4], and James A. Coffman[1,2]*

[1]MDI Biological Laboratory, Salisbury Cove, Maine 04672, USA

[2]Graduate School of Biomedical Science and Engineering, University of Maine, Orono, Maine 04469, USA

[3]The School of Marine Sciences, University of Maine, Orono, ME 04469, USA

[4]Nicholas School of the Environment, Duke University, Durham, NC, 27708.

*Correspondence: jcoffman@mdibl.org

Supplementary Methods

Zebrafish husbandry and treatments

Zebrafish were maintained in the animal facility of the MDI Biological Laboratory, in a recirculating system with a water temperature of 28.5°C, conductivity of 600-700 microsemens, pH of 7.2. Lights are timed on a 14 h light/10 h dark cycle. Matings and embryo/larvae culture were carried out using standard procedures [1]. Spawning tanks were set up overnight to allow fish to acclimate, with male and female fish separated by a divider. The following morning dividers were removed, and animals allowed to spawn. Embryos were collected in the late morning or early afternoon and disinfected for one minute with peroxy-acetic acid solution consisting of 60ul concentrated Peroxy-Serve (Axela Medical) per 40ml of E2 media, followed by two 2-minute rinses in fresh E2 media. Embryos were then transferred to petri dishes containing 40ml of fresh E2 media at a density of 100 embryos per plate or fewer.

For cortisol treatment, stock solutions of cortisol-21-hemisuccinate sodium salt (Sigma H4881) in DMSO were added to the medium to achieve 1uM concentration. For vehicle control treatment an equivalent concentration of DMSO alone was used. Cortisol or vehicle treatments began ~4 hours post-fertilization. Embryos/larvae were cultured in a 28.5C incubator on a light–dark cycle (14 h light–10 h dark) synchronized with the fish core room. Cortisol and control media were refreshed daily. For experiments using FK506 an overnight exposure (17 hours) to either 1uM drug or vehicle control was started on the afternoon of day 4 post-fertilization.

Klf9-AM-tag CRISPR line

DNA templates for guide RNAs (sgRNA) and homology directed repair (HDR) were synthesized using fusion PCR with Taq polymerase (NEB) and oligonucleotides from IDT. Templates were run through agarose gel to confirm correct size. HDR template was extracted using EZNA Gel Extraction Kit (Omega Biotek) and confirmed by Sanger sequencing. HDR template sequence consisted of 99bp AM-Tag (Active Motif) sequence inserted in-frame prior to endogenous stop codon and flanked by ~60bp micro-homology arms. Silent and PAM mutations were designed into HDR template to prevent editing of

template by CRISPR. Two sgRNA were designed using online software at Benchling.com, synthesized from templates using MegaScript T7 Transcription Kit (ThermoFisher), and purified by phenol:chloroform extraction. Fertilized AB strain embryos were injected at the one-cell stage with a CRISPR mix containing the following: 200ng/µl Cas9 mRNA, 100ng/µl sgRNA, 200ng/µl HDR template, 0.1M KCl, and 0.05% Phenol Red dye. F0 mutants were identified by fin-clip genotyping and outcrossed to AB strain fish. F1 heterozygous mutants were identified by fin-clip genotyping and in-crossed to produce embryos for chromatin immunoprecipitation experiments.

Cortisol measurement

Samples for basal cortisol measurements were plated individually during the daily media change on 4 dpf (i.e., the day before sampling occurred). Individual plates of pooled larvae (n=15-20 per sample) were pulled from incubator, media were removed, and samples were snap frozen in liquid nitrogen in less than two minutes, then stored at -80C for later processing. Each sample was collected with minimal disturbance to other plates in the incubator. During collection of samples before "lights-on" in the incubator, room lighting was kept to a minimum.

For acute stress cortisol measurements, larvae were left undisturbed overnight in incubator. On 5 dpf, larvae were chased and captured with a transfer pipette at time=0 (~3 ZT). T=0 samples were snap frozen immediately, and the rest were transferred to separate plates for subsequent snap freezing at 10-, 20-, 30- and 60-minutes post-chase. Each sample consisted of a pool of n=14-18 larvae.

Frozen samples were homogenized in PBS by motorized pestle. Two rounds of liquid-liquid extraction were performed with ethyl acetate and organic phase collected. Solvent was evaporated and residue re-dissolved in extraction buffer (Neogen Corporation). Cortisol was measured using a PowerWave XS plate reader (Bio-Tek) and quantified with a standard curve according to the manufacturer's protocol (Neogen Corporation).

Quantitative reverse transcription and polymerase chain reaction (qRT-PCR)

Larvae were snap frozen in liquid nitrogen and total RNA was extracted and purified using TRIzol (ThermoFisher) according to manufacturer's instructions. Quality and yield of RNA were measured using a DeNovis DS-11 FX spectrophotometer. cDNA was synthesized using Primescript cDNA kit (TaKaRa). Relative gene expression levels were measured by qRT-PCR, using SYBR Green FastMix (Quanta Bio) and a Roche Lightcycler 480II instrument. Ten microliter reactions consisted of 5ul FastMix, 1ul 3uM primers, and 4ul diluted cDNA. The delta-delta Ct method was used to calculate relative expression of transcripts using *eif5a* as a reference gene. Examination of the results of multiple prior RNA-seq data sets indicated that *eif5a* activity was highly stable across treatments and genotypes. In many experiments beta-actin and/or *rpl13a* were also used as a reference gene, and this did not substantially change the results. For time-course experiments, relative expression of gene of interest across time was calculated relative to the median expression level of the gene of interest in all samples in that experiment. For primers see Table S1. Each sample for 5dpf time-course experiments was plated individually during daily media change on 4dpf, and samples were collected with minimal disturbance to other samples in the

incubator. Samples frozen prior to ZT 0 (lights-on) were collected from the incubator with minimal room lighting.

Nanostring analysis

Nanostring probes are made with target specific sequences and tag specific sequences at 5' and 3' tailing ends. The selected probe sets made by Integrated DNA Technologies, Coralville, Iowa, were mixed and diluted into pools then combined with one of the 8 unique barcoded PlexSets.

NanoString assays were performed by Dartmouth College's Molecular Biology Core Facility. The probes and PlexSet pools are combined and hybridized at 67 °C for 18 h with their RNA targets, with a distinct PlexSet in each 8 wells of a column in a 96 well plate. Each of the 12 columns from the 96-well plate was then pooled for processing on the NanoString nCounter Prep Station. The Prep Station purifies the target/probe complexes and deposits them in a cartridge where they are immobilized and aligned for data collection. Data Collection is carried out in the NanoString nCounter Digital Analyzer. Images are processed and an algorithm tabulates digital counts for each barcode class.

An initial RNA titration test was performed for each probe set with 50 ng, 100 ng, and 200 ng RNA to optimize the RNA concentration. 100 ng was selected for the full-time course assay.

RNA-seq and data analysis

The RNA-seq experiment that generated the data described here was described in a previous publication [2]. Briefly, four replicates of n=10 larvae were snap frozen in each condition, and RNA-extracted using the Qiagen RNA-Easy Plus Mini Kit. RNA extraction was done in two batches on different days. On the first day (experimental replicates 1 and 2) the lysis buffer was added to all 8 frozen samples on ice before homogenization, while on the second day the lysis buffer was added to each sample which was then immediately homogenized. This difference in sample preparation likely accounts for a significant portion of variance between samples prepared on day 1 and day 2, and a batch effect was included as a two-level categorical covariate in determining differential expression (see below).

RNA-seq libraries were generated with Illumina-compatible KAPA libraries and sequenced on an Illumina NextSeq 500 High Output sequencer. *klf9*$^{-/-}$ and matched control samples were sequenced as single end 75-bp reads. VBA+ and VBA- samples were sequences as paired-end 75-bp samples.

Fastq formatted read files were preprocessed with Trimmomatic version 0.38 [3] with default options, and then aligned to the Zebrafish genome version 11 as presented in ENSEMBL version 93, using the STAR aligner version 2.6.1b [4]. The Ensembl transcriptome was preprocessed with a splice junction overhang of 100 nt. Following alignment, the resulting BAM files were processed with RSEM version 1.3.0 [5] for isoform and gene-level expression estimates. The resulting gene-level expression values were merged into a single expression matrix with an in-house python script. EDASeq [6] carried out in R version 3.6.1 was used to further normalize data for systematic effects, using gene-level length and GC-content as downloaded from Ensembl version 98 using EDAseq's included scripts. "WithinLane" normalization with GC content was judged as superior to that based on length. Final normalized gene-

level counts (which = “full”) were generated using GC-based WithinLaneNormalization followed by BetweenLaneNormalization. Differential expression analysis was carried out in R version 3.6.2 with DESeq2 [7] version 1.26.0, using genotype or treatment as the comparison.

The GOrilla algorithm [8] (https://cbl-gorilla.cs.technion.ac.il/) was used for Gene Ontology term enrichment analysis, and the data were visualized using REVIGO [9] (https://revigo.irb.hr/), with box size corresponding to fold enrichment. HOMER motif enrichment analysis [10] was used to compare incidence of known vertebrate motifs in a list of promoters of interest with incidence in a background list of all zebrafish promoters by running the findMotifs program (https://homer.ucsd.edu/homer/microarray/index.html) using default settings except that sequence from − 2000 to + 2000 bp relative to the transcription start site was searched for motifs from 10 to 14 bp in length. KEGG pathway analysis was performed using the DAVID Bioinformatics Resources website [11,12] (https://david.ncifcrf.gov/), comparing list of differentially expressed genes against species specific background. For principal component analysis of metabolic genes of interest (Table S3) Z scores were generated using log transformed RNA-seq counts. Z scores were calculated within batches (RNA preparation day 1 and day2, see above) and scores were then compiled. PCA was then run using PCAtools version 1.2.0 in R version 3.6.2.

Chromatin immunoprecipitation (ChIP)

DynaProtein A beads (Invitrogen) were blocked by washing 3X in blocking solution (0.5% BSA in PBS). Dyna beads were then incubated with antibody (anti-acetyl-H3K14, Active Motif cat# 39698; anti-Klf9, ABCam ab227920; non-specific IgG negative control, Invitrogen 02-6102; or anti-H3K4me3 ABCam ab8580, as positive control) in blocking solution on a rotator at 4° C overnight.

DNA-protein crosslinking and chromatin preparation

Whole 5dpf larvae (60-80 per replicate) were euthanized with Tricaine (Sigma) and immediately incubated for 15 minutes on a room-temperature rotator in 1.85% formaldehyde in PBS to crosslink protein and DNA. Formaldehyde was then quenched with glycine added to a concentration of 0.125M for 5 minutes. Larvae were then rinsed three times in cold PBS, pelleted by centrifuging briefly and either frozen at -80° C or immediately homogenized and lysed. Homogenization was performed with a motorized micro-pestle in cell lysis buffer (10mM Tris-HCl, pH 8; 10mM NaCl, 0.5% IGEPAL) with protease inhibitor (cOmplete). Lysing suspension was kept on ice and homogenized periodically for 15 minutes. Nuclei were then pelleted in a cold centrifuge, supernatant removed, and pellet resuspended in nuclei lysis buffer (50mM Tris-HCl, pH8; 10mM EDTA, 1% SDS) with protease inhibitor. Nuclei lysis was incubated on ice for 10 minutes. Two volumes of IP dilution buffer were then added (16.7mM Tris-HCl pH 8, 167mM NaCl, 1.2mM EDTA, 0.01% SDS, protease inhibitor). Chromatin (<1ml in volume) was sheared into fragments using a QSonica Q500 sonicator and microtip probe. Samples were sonicated on ice, with a series of 10-second pulses at 20% amplitude (lowest setting, to avoid foaming) separated by 20-second cooling intervals. During sonication, progress was monitored by running 5ul of sample on a 1% agarose gel with SybrSafe Dye (Invitrogen). Bands of sheared, crosslinked DNA at 800-1000bp were observed after 50-60 pulses, and subsequently determined to correspond to DNA fragments of <500bp

(assayed again on 1% agarose gel after reversal of crosslinking, see below). 48ul of 10% Triton-X (Fisher) were added per 0.6ml of sonicated sample, and samples were then centrifuged at 4° C at max speed for 15 minutes. A small volume of supernatant (e.g. 50ul) was saved as input control and the remaining supernatant was incubated with antibody-coated beads overnight at 4° C on a rotator.

Washing, elution, and reversal of crosslinks

Beads were spun down and collected with magnetic stand, and supernatant was removed. Beads were washed five times with RIPA buffer (50mM HEPES pH8, 1mM EDTA, 0.7% sodium-deoxycholate, 1% IGEPAL, 0.5M LiCl), spun down and collected by magnetic stand after each wash. Beads were washed with TBS, spun down and TBS was aspirated. DNA was eluted from beads in elution buffer (50mM $NaHCO_3$, 1% SDS) at 65° C for 15 minutes with periodic vortexing. Beads were then spun down at max speed and supernatant transferred to a new tube. Four volumes of elution buffer were added to input control samples, and 5M NaCl was added to all samples to final concentration of 0.2M before incubation overnight at 65° C to reverse formaldehyde crosslinks.

Precipitation and DNA Purification

RNAse A was added to a final concentration of 0.33ug/ul, and samples were incubated at 37° C for 2 hours. Samples were chilled briefly before proteinase K was added to 0.2ug/ul and incubation at 65°C for 2 hours. After incubation, samples were chilled on ice and 1 volume of phenol/chloroform/isoamyl alcohol was added. Samples were mixed and spun at high speed for 5 minutes in cold centrifuge. Aqueous layer was transferred to a new tube and 20ug glycogen added. One tenth volume of 3M NaOAc and two volumes of 100% EtOH were added. Samples were mixed and then spun at top speed for 30 minutes in cold centrifuge to pellet DNA. Supernatant was removed and pellets washed twice in 75% EtOH. Pellets were briefly air dried and then resuspended in 10mM Tris-HCl, pH 8. DNA concentration was assayed on a DeNovix spectrophotometer.

Quantitative PCR (qPCR)

For qPCR analysis, serial dilutions of input control DNA were used to generate standard curves covering the range of ChIP DNA samples. Input and immunoprecipitated samples were quantified in 10ul qPCR reactions consisting of 5ul SybrGreen FastMix (Quanta Bio), 1ul 3uM primers, and 4ul of diluted DNA template. Primers were designed to target a putative Klf9 binding site in the *fkbp5* promoter (F: ccaaggcctgcccttaattt, R: cctctgcgagacatttggac). Concentration of ChIP DNA was calculated from standard curve and converted to percent recovery ([ChIP DNA] / [input control DNA])*100.

<u>Oxygen Consumption Rate</u>

Measurements were made per single 1dpf embryo transferred with 150 uL of either (a) egg water + DMSO vehicle (VEH group) or (b) egg water + 1 micromolar cortisol (CORT group), contained in an individual well in a spheroid microplate (Agilent Technologies, CA). Each embryo was centered in the spheroid chamber in the bottom of the well and air bubbles were removed. Seven-minute basal measurement cycles, consisting of a 2:00 min mix, 2:00 min wait, and 3:00 min measure period, were

collected over a course of at least 85 minutes (corresponding to 12 measurement cycles). A total of 60 individual embryos per treatment group was tested. Embryo specific OCR per treatment were normalized to background wells containing egg water + DMSO vehicle (VEH group) or (b) egg water + 1 micromolar cortisol (CORT group).

References

[1] A. Nasiadka, M.D. Clark, Zebrafish breeding in the laboratory environment, Ilar J 53 (2012) 161-168.
[2] I. Gans, E.I. Hartig, S. Zhu, A. Tilden, L. Hutchins, N. Maki, J.H. Graber, J.A. Coffman, Klf9 is a key feedforward regulator of the transcriptomic response to glucocorticoid receptor activity, Sci Rep 10 (2020).
[3] A.M. Bolger, M. Lohse, B. Usadel, Trimmomatic: a flexible trimmer for Illumina sequence data, Bioinformatics 30 (2014) 2114-2120.
[4] A. Dobin, C.A. Davis, F. Schlesinger, J. Drenkow, C. Zaleski, S. Jha, P. Batut, M. Chaisson, T.R. Gingeras, STAR: ultrafast universal RNA-seq aligner, Bioinformatics 29 (2013) 15-21. 10.1093/bioinformatics/bts635.
[5] B. Li, C.N. Dewey, RSEM: accurate transcript quantification from RNA-Seq data with or without a reference genome, BMC Bioinformatics 12 (2011) 323.
[6] D. Risso, K. Schwartz, G. Sherlock, S. Dudoit, GC-content normalization for RNA-Seq data, BMC Bioinformatics 12 (2011) 480. 10.1186/1471-2105-12-480.
[7] M.I. Love, W. Huber, S. Anders, Moderated estimation of fold change and dispersion for RNA-seq data with DESeq2, Genome Biol 15 (2014) 550. 10.1186/s13059-014-0550-8.
[8] E. Eden, R. Navon, I. Steinfeld, D. Lipson, Z. Yakhini, GOrilla: a tool for discovery and visualization of enriched GO terms in ranked gene lists, BMC Bioinformatics 10 (2009) 48.
[9] F. Supek, M. Bosnjak, N. Skunca, T. Smuc, REVIGO summarizes and visualizes long lists of gene ontology terms, PLoS One 6 (2011) e21800.
[10] S. Heinz, C. Benner, N. Spann, E. Bertolino, Y.C. Lin, P. Laslo, J.X. Cheng, C. Murre, H. Singh, C.K. Glass, Simple combinations of lineage-determining transcription factors prime cis-regulatory elements required for macrophage and B cell identities, Mol Cell 38 (2010) 576-589.
[11] W. Huang da, B.T. Sherman, R.A. Lempicki, Systematic and integrative analysis of large gene lists using DAVID bioinformatics resources, Nat Protoc 4 (2009) 44-57.
[12] W. Huang da, B.T. Sherman, R.A. Lempicki, Bioinformatics enrichment tools: paths toward the comprehensive functional analysis of large gene lists, Nucleic Acids Res 37 (2009) 1-13. 10.1093/nar/gkn923.
